# Patient reported outcomes in pediatric physical therapy: a scoping review and evidence map

**DOI:** 10.1186/s41687-025-00947-5

**Published:** 2025-10-24

**Authors:** Dorinde L. Korteling, Selina Limmen, Marjolijn Ketelaar, Joost G. Daams, Michiel A. J. Luijten, Hedy A. van Oers, Manon A. T. Bloemen, Lotte Haverman, Raoul H. H. Engelbert

**Affiliations:** 1 Child and Adolescent Psychiatry & Psychosocial Care, Amsterdam UMC Location University of Amsterdam, Emma Children’s Hospital, Amsterdam, The Netherlands; 2 UMC Utrecht Brain Center, University Medical Center Utrecht, Utrecht, The Netherlands; 3 Center of Excellence for Rehabilitation Medicine Utrecht, De Hoogstraat Rehabilitation, Utrecht, The Netherlands; 4 Medical Library, Amsterdam UMC Location University of Amsterdam, Amsterdam, The Netherlands; 5 Research Group Moving, Growing and Thriving Together, HU University of Applied Sciences Utrecht, Utrecht, The Netherlands; 6 Centre of Expertise Urban Vitality, Faculty of Health, Amsterdam University of Applied Sciences, Amsterdam, The Netherlands; 7 Child Development, Amsterdam Reproduction and Development, Amsterdam, The Netherlands; 8 Mental Health, Amsterdam Public Health, Amsterdam, The Netherlands; 9 Methodology, Amsterdam Public Health, Amsterdam, The Netherlands; 10 Personalized Medicine, Amsterdam Public Health, Amsterdam, The Netherlands; 11 Department of Epidemiology and Data Science, Amsterdam UMC, Vrije Universiteit, Amsterdam, The Netherlands; 12 Quality of Care, Amsterdam Public Health, Amsterdam, The Netherlands; 13 Digital Health, Amsterdam Public Health, Amsterdam, The Netherlands; 14 Department of Rehabilitation Medicine, Amsterdam Movement Sciences, Amsterdam UMC, University of Amsterdam, Amsterdam, The Netherlands

## Abstract

**Background:**

Patient-reported outcome measures (PROMs) can be used as tools for understanding patients’ health perceptions. Gaining a comprehensive understanding of the landscape of PROs and PROMs within PPT, along with identifying potentially valuable (generic) PROs for the specific context and population, provides a valuable foundation for developing recommendations on PROM use in PPT.

**Objectives:**

To provide a scoping review of measured PROs in studies describing PPT interventions. Additionally, to provide an overview of used PROMs in PPT.

**Methods:**

This review is conducted based on the PRISMA-ScR Checklist. A systematic search was conducted in Medline (2013–2023). Peer-reviewed studies for children aged 4–17 years with problems in physical functioning, were included when a described intervention was related to PPT or exercise therapy, and the studies reported PROs or used PROMs (proxy/self-reported). PROs were extracted verbatim, categorized and labelled based on the Alonso & Valderas model. A graphical overview was created to synthesize PROs measured per diagnosis.

**Results:**

172 studies were included. We identified 168 measured PROs which could be categorized into 40 unique PROs, measured with 158 PROMs. Most measured PROs fell in the ‘Functional Status – Activities and Participation’ and ‘Symptom Status’ classification of the Alonso and Valderas model.

**Conclusion:**

An excessive number of PROs and PROMs is used in PPT, which complicates standardization and implementation of PROMs. Harmonization of PRO(M)s in PPT is needed to address this problem. Therefore, a generic core set of PROs and PROMs should be developed for daily practice and research within PPT.

**Supplementary Information:**

The online version contains supplementary material available at 10.1186/s41687-025-00947-5.

## Introduction

Pediatric physical therapy (PPT) addresses movement-related problems in children, such as developmental issues, child-specific pathologies, and conditions originating in childhood [1]. PPT focuses on helping children achieve their maximum potential for independent functioning and active participation at home, school, and in community environments by collaborating with children and their families [1, 2]. Working across different care settings, pediatric physical therapists apply clinical reasoning during assessment, intervention, and evaluation [2]. Clinical measurements, such as mobility and motor skills, are often emphasized in PPT [3–5]. While these measurements are essential for PPT interventions, they do not encompass the full multidimensional aspects of children’s and their caregivers’ health perspectives [4, 6, 7]. The American Physical Therapy Association Guide to Physical Therapist Practice recommends using outcome measures across all domains of the International Classification of Functioning, Disability and Health (ICF) [3]. This ensures a comprehensive approach that addresses not only physical abilities but also the broader multidimensional aspects of health and well-being for children and their caregivers, recognizing that various health factors can influence children’s independent functioning and active participation.

Recommendations to incorporate the multidimensional health perspectives of children and their caregivers into PPT interventions have led to a growing recognition of the importance of patient reported outcome measures (PROMs) as tools for assessing and monitoring patient reported outcomes (PROs). PROs are outcomes directly reported by the patient or a proxy (such as caregiver), without interpretation by a healthcare professional or others. They relate to the patient’s health perception, functional status, or quality of life, associated to their healthcare intervention, and can be measured with PROMs [8, 9]. Psychometrically reliable and valid PROMs offer insights into patient perceptions about their health, and are meant to be used alongside other clinical outcome assessments such as performance tests. PROMs incorporated in healthcare contribute to personalized interventions and improved patient-centered care by facilitating more structured and in-depth communication [10–12], supporting shared decision-making [13, 14] and guiding goal setting [4, 15, 16]. Additionally, PROMs can ensure that patients are more prepared for a consultation [10, 11].

Recognizing their potential benefits, the use of PROMs has gained prominence in healthcare [17], and is widely recommended by national health authorities [18]. Although the importance of PROMs is recognized in PPT, different studies stated difficulties in selecting valid and reliable PROMs for PPT [4–6, 12]. Several factors, identified in PPT field and healthcare in general, may contribute to these difficulties [4–6, 12, 19, 20]. First, different PRO terminology complicates comparison or standardization of PRO data, and reduces clarity among studies [19]. For instance, the PRO ‘physical function’ may also be referred to as ‘activities of daily living’, ‘activity limitations’, or ‘mobility’. Second, PROMs can be generic, measuring a PRO relevant for many patients, or disease-specific, measuring a PRO relevant to a specific group of patients [20]. PPT addresses a heterogeneric population in terms of age, condition, level of functioning, and participation, as well as care setting, which may contribute to the belief that different PROMs are necessary for different patient populations. However, there is significant overlap in generic PROs within disease-specific PROMs, mainly because the same PROs are often important for different patient populations. Therefore, various expert workgroups stated the importance of using generic PROMs where possible, and to supplement them with disease-specific PROMs as needed [19, 21, 22]. Third, the multitude of PROMs available, partly explained by the above mentioned reasons, may lead to difficulties in choosing internationally acknowledged, valid and reliable PROMs [19]. This is exemplified by a study amongst pediatric physical therapists, where uncertainty regarding the appropriateness of measures and lack of familiarity with measures were identified as a barriers to PROM use [12].

Gaining a comprehensive understanding of the landscape of PROs and PROMs within PPT, along with identifying potentially valuable (generic) PROs for the specific context and population, provides a valuable foundation for developing recommendations on PROM use in PPT [23]. Therefore, the aim of this study is to provide a scoping review and evidence map of measured PROs in the last decade (2013–2023), used as outcomes of PPT studies for children and adolescents 4 to 17 years of age, with problems in physical functioning. Additionally, this study will provide an overview of used PROMs to measure these PROs. The results of this study can guide future recommendations for PROM use in PPT, ultimately benefitting structural incorporation of multidimensional health perspectives of children and their caregivers in daily care.

## Methods

This scoping review and evidence map were drafted according to the Preferred Reporting Items for Systematic reviews and Meta-Analyses extension for Scoping Reviews (PRISMA-ScR) Checklist [24]. The protocol has been published beforehand in the Open Science Framework: “Patient Reported Outcomes measured in Pediatric Physical Therapy: An Evidence Map”, protocol number wv89k (https://osf.io/wv89k/wiki/home/).

### Eligibility criteria

Peer-reviewed studies involving children aged 4–17 years with limitations in physical functioning (defined as problems in the child’s ability to move independently, motor development, and active participation in daily life) [2], were included when a described intervention (1) was related to PPT or exercise therapy (including different healthcare settings such as primary care or rehabilitation), and (2) reported PROs or used self-reported PROMs or PROMs proxy-reported by caregivers. We excluded studies only focusing on children under four years old, as outcomes might significantly differ for this age group compared to older children, and observer reported outcomes may be more suitable. Furthermore, we excluded study-protocols, literature reviews, reports with no original data, papers in which the role of the (pediatric) physical/exercise therapist in the intervention process was not described, and papers not written in English or Dutch.

### Search strategy

For this review, Medline was searched. Guided by a medical information specialist, a search strategy was determined. Key search concepts were ‘physical therapy’, ‘child’, ‘patient reported outcome’ and ‘patient reported outcome measure’. A visualization of similarities (VOS) analysis, using VOSviewer [25], was used to specify the search strategy, by identifying possible irrelevant concepts to be precluded from the search (e.g. ‘Law’ and ‘Smoking’). Given the steep increase in academic literature regarding PROMs since 2013 [26], this review was limited to articles published from 2013 onwards. This timeframe coincides with global shifts in pediatric physical therapy highlighted by the World Health Organization’s 2013 and 2014 action plans, from treatment of the medical diagnosis towards emphasizing physical functioning with related societal participation of children with health issues [1, 27–29].

The full search strategy is provided as supplementary data (Additional file 1).

### Selection of sources of evidence

All studies identified by the search were imported into an in-house developed tool for removal of duplications. De-duplicated studies were uploaded into the web application Rayyan (https://www.rayyan.ai/), after which they were screened for potential inclusion based on title and abstract [24]. 10% of studies were screened by two researchers (DK and SL) and two research assistants (TS and LV) in collaboration. The remaining 90% of titles and abstracts were screened by two researchers (DK and SL) or two research assistants (TS and LV) independently. If the title and abstract did not provide sufficient information to judge the record for relevance, the full-text article was assessed for eligibility.

All articles deemed eligible based on title and abstract were screened based on full-text according to pre-defined selection criteria for inclusion in the review. This was performed by two researchers independently (DK and SL).

In all stages of the screening process, discrepancies between researchers were discussed until consensus was reached. In cases where agreement could not be achieved, articles were labeled and set aside. Subsequently, a third researcher (ML) was consulted in a live meeting with both DK and SL, during which the articles were discussed until consensus was reached.

### Data extraction

Two researchers (DK and SL) developed a data-charting form to identify variables to be extracted. Data extraction comprised the following topics: general information (e.g. author(s), year of publication, study design, and number of participants), demographics (e.g. age, sex), intervention type (e.g. multidisciplinary, home rehabilitation), diagnosis (e.g. musculoskeletal disorders, neurological disorders), PRO (e.g. physical functioning, anxiety, social functioning), and PROM (e.g. Patient-Reported Outcomes Measurement Information System^®^ (PROMIS^®^) or Pediatric Quality of Life Inventory (PedsQL™ 4.0)). Additionally, researchers noted if the used PROM was generic or disease-specific. For extraction of PROs, researchers identified PROs directly described in the paper or, if absent, extracted them from the utilized PROMs. This was done by identifying the domains or subscales measured by the PROM (e.g. PedsQL™ 4.0 consists of four subscales: physical functioning, emotional functioning, social functioning and school functioning).

### Synthesis of results

Both PROs and diagnoses were categorized to be able to provide a clear overview of the results. Categorization of PROs (DK) and diagnoses (SL) was performed by one researcher independently, after which the results were discussed in depth. When no agreement was found a third researcher within the team was consulted (RE or MK). The final categorizations were discussed within the research group, including pediatric physical therapists, pediatric psychologists and PROM experts. Sociodemographic information of all included studies was aggregated to provide a comprehensive overview.

### Diagnoses

Medical diagnoses, and/or reasons for physical therapeutic treatment, extracted from the articles were categorized for a clear overview in a graphical format. Initially, diagnoses/reasons for treatment were classified as either ‘chronic’ (defined as a diagnosis of which the course lasts for more than three months) or ‘acute’ (defined as a diagnosis of which the course lasts for less than three months) [30]. Subsequently, they were further categorized as: (1) Musculoskeletal; (2) Neurological; (3) Gastrointestinal, endocrine and hematological; (4) Cardiovascular and respiratory; (5) Oncology or (6) Mental health.

### PROs

To organize the verbatim reported PROs, the research group categorized them according to two internationally acknowledged frameworks: ICF [3] or the PROMIS^®^ conceptual framework [31]. The ICF is a classification system of health status that considers the full spectrum of disability [3]. PROs were initially matched with terminology from the ICF browser (https://apps.who.int/classifications/icfbrowser/) by using the most specific category within the ICF that matched the PRO’s definition [32]. For example, the PRO ‘bimanual activities’ was categorized as ‘hand and arm use’ within the ICF. As the ICF does not address mental health concepts on a detailed level, it was supplemented by the PROMIS^®^ conceptual framework, a guide for the development of distinct PROs about social, mental and physical health [31]. For example, the PROMIS^®^ conceptual framework was utilized for PROs within the domain of mental health, such as anger, depression, or anxiety.

**Fig. 1:**
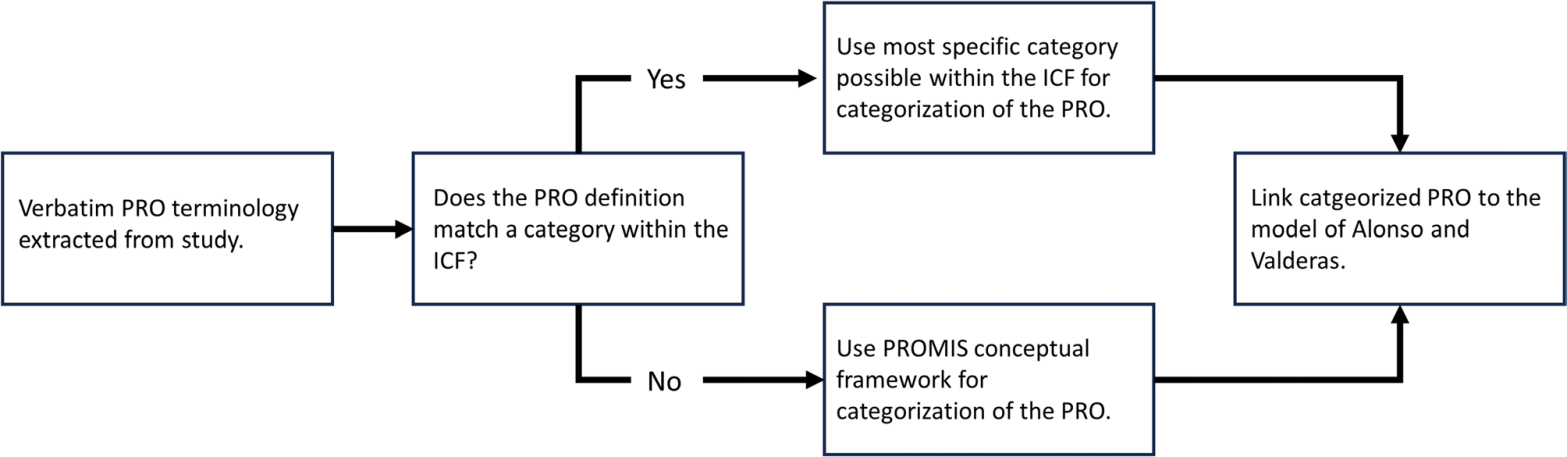
Flowchart of the procedure of Patient Reported Outcome (PRO) categorizing. PROs were categorized within the International Classification of Functioning, Disability and Health (ICF) or the Patient-Reported Outcomes Measurement Information System^®^ (PROMIS^®^) conceptual framework, after which they were placed within the Alonso and Valderas model [33]

To provide a structured overview of PROs within a graphical format, the categorized PRO terminologies were labelled according to the Alonso & Valderas model [33], a classification system of PRO measures. Figure 1 shows a flowchart of the procedure of PRO categorizing. The Alonso & Valderas model combines the ICF and the Wilson and Cleary model [34], a bio-psycho-social model for health outcomes. To create more nuances within the classification ‘Functional Status’ of the Alonso & Valderas model, sub-categories ‘Social Health’ and ‘Mental Health’ were incorporated based on the PROMIS^®^ conceptual framework, in alignment with research performed by Oude Voshaar et al. [21]. Additional file 2 contains detailed information about the Alonso & Valderas model and its adaptations for this study.

### PROs per diagnosis

PROs measured within each diagnostic group were described and presented in an evidence map. An evidence map can be understood as a systematic (visual) presentation of the available evidence regarding a certain topic [35]. The most frequently measured PROs across all diagnostic groups, as well as patterns specific to individual groups, were examined and reported.

### PROMs

All PROMs used across the included studies were extracted and categorized as either generic or disease-specific. The number of unique PROMs used to assess each PRO was recorded. Descriptive statistics, including mean, standard deviation, and range, were calculated to quantify the number of PROMs used per PRO.

## Results

**Fig. 2:**
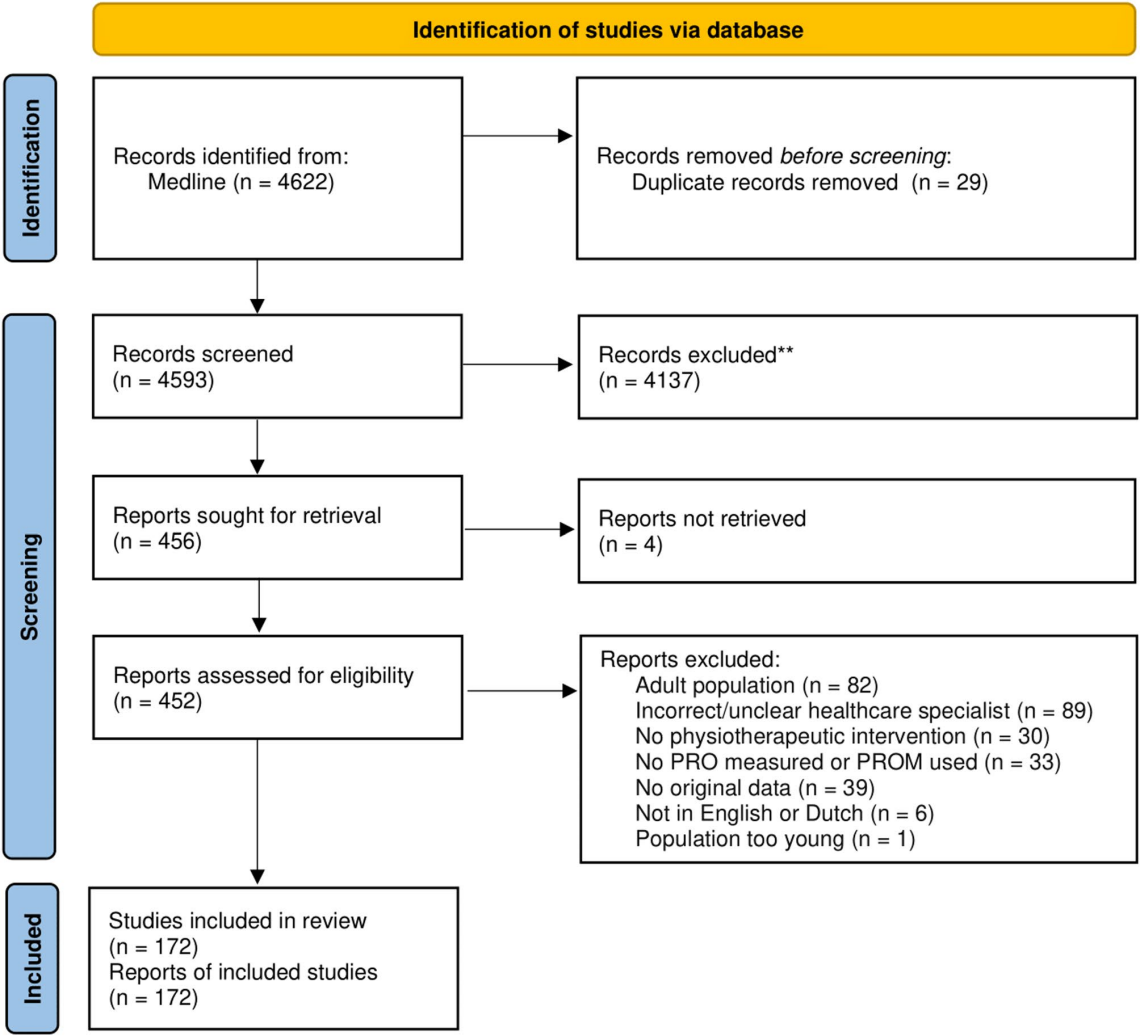
PRISMA 2020 flow diagram, according to the preferred reporting items for systematic reviews and meta-analyses extension for scoping reviews (PRISMA-ScR) Checklist [24]

The search in Medline yielded 4622 studies (see PRISMA flow diagram; Fig. 2). After deduplication, 4593 studies remained. Screening of title and abstract yielded 456 studies, of which 452 were assessed based on full-text for eligibility. For the remaining four studies, full-text was not available. 172 studies met the inclusion criteria (references can be found in Additional file 3), which reported in total on 11,769 patients. Sociodemographic information can be found in Table 1. Of all included studies, 94 (54.7%) reported on multidisciplinary healthcare interventions, with one of the disciplines being PPT. Thirty-two (18.6%) studies reported on home rehabilitation guided by a physical therapist, in some cases virtually or over the phone. Twenty-nine (16.8%) studies reported on children as well as adults.

**Table 1:** Sociodemographic information of all 172 included studies, which reported on 11,769 patients

**Sociodemographic information**	**All included patients (** ***n*** ** = 11,769)**
Gender	
Boy	3540 (30.1%)
Girl	5842 (49.6%)
Non-binary	1 (0.008%)
Not reported	2386 (20.1%)
**Sociodemographic information**	**All included studies (** ***n*** ** = 172)**
Number of studies including participants within a specific age range*	
4–7 years	98
8–11 years	124
12–15 years	130
16–17 years	92
Number of studies reporting on physical therapeutic intervention with a specific duration*	
1 to 2 weeks	9 (5.2%)
2 to 4 weeks	25 (14.5%)
1 to 2 months	33 (19.2%)
2 to 3 months	31 (18.0%)
3 to 6 months	46 (26.7%)
6 months to 1 year	18 (10.1%)
1 year or more	4 (2.3%)
Not reported	7 (4.1%)
Number of studies reporting on acute or chronic condition / diagnoses	
Acute	36 (20.9%)
Chronic	136 (79.1%)
Number of studies reporting on type of medical diagnosis and/or reason for physical therapeutic treatment*	
Musculoskeletal	62 (35.2%)
Neurological	84 (47.7%)
Gastrointestinal, endocrine and hematological	10 (5.7%)
Cardiovascular and respiratory	9 (5.1%)
Oncology	7 (4.0%)
Mental Health	4 (2.3%)

*As some studies included participants across multiple age ranges, varied durations of physical therapeutic interventions or multiple types of diagnoses, the total of these numbers exceeds the total number of included studies (*n* = 172). To avoid confusion, percentages have been left out for the number of studies including participants within a specific age range

### Diagnoses

The included studies reported on children with 76 different medical diagnoses and/or reasons for physical therapeutic treatment, which were grouped within 6 categories (Musculoskeletal (35.2%); Neurological (47.7%); Gastrointestinal, endocrine and hematological (5.7%); Cardiovascular and respiratory (5.1%); Oncology (4.0%); Mental health (2.3%); Table 1). The most common medical diagnosis was cerebral palsy (49 studies; 28.5%). Most of the diagnoses could be considered chronic (79.1%) rather than acute.

### PROs

In total, 168 reported PROs were extracted verbatim from the 172 included studies. All 168 reported PROs could be categorized into 40 unique PROs. Of these 40 PROs, 37 covered terminology from the ICF and 3 covered terminology from the PROMIS^®^ conceptual framework. The most commonly measured PROs fell within the categories pain (14.8%), overall quality of life (12.1%), general tasks and demands (7.6%), and neuromusculoskeletal and movement-related functions (7.6%), and goal attainment (6.0%). Within the Alonso & Valderas model, most measured PROs could be linked to the ‘Functional status – Activities and Participation’ classification (28.6%) and the ‘Symptom Status’ classification (22.4%).

### PROs per diagnosis

**Fig. 3:**
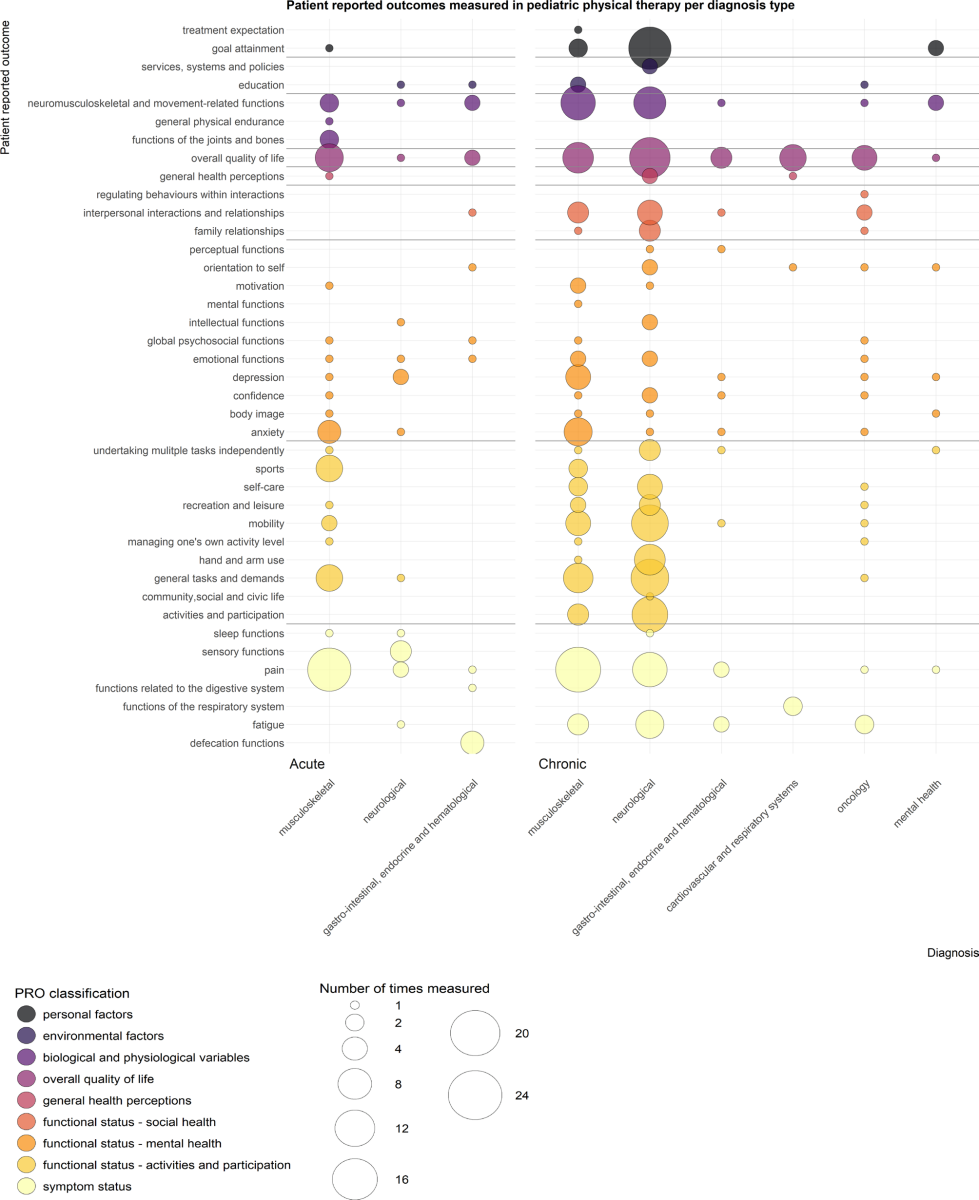
Evidence map of Patient Reported Outcomes (PROs) measured in pediatric physical therapy per diagnosis type. The size of the circles indicates the number of the respective PRO measured within a population with a specific diagnosis. The color of the circles indicate the PROs classification based on the Alonso & Valderas model.

As illustrated in the resulting evidence map (Fig. 3), the PRO classifications ‘Overall Quality of Life’, ‘Functional Status – Mental Health’, and ‘Symptom Status’ were measured across all of the diagnostic groups. The following PRO classifications were measured across at least three-quarter of the diagnostic groups: ‘Biological and Physiological Variables’ (88.8% of diagnostic groups) and ‘Functional Status – Activities and Participation’ (77.7% of diagnostic groups).

No clear pattern between measured PROs and diagnostic groups emerges from the evidence map (Fig. 3). As such, we highlight the main findings for the most frequently measured PRO classifications (‘Functional status – Activities and Participation’ and ‘Symptom Status’). 55% of PROs labelled as ‘Functional Status – Activities and Participation’ within the Alonso & Valderas model were measured within children with chronic neurological diagnoses. 51% of PROs labelled as ‘Symptom Status’ within the Alonso & Valderas model (including pain) were measured in children with musculoskeletal diagnoses.

### PROMs

In the 172 included studies 158 different PROMs were used, of which 92 (58.2%) were generic and 66 (41.8%) disease-specific. The table in which the PROMs are reported can be found in Additional file 4. On average, 17.4 PROMs (standard deviation: 18.96; range: 2–78) were used to measure a single PRO. The number of PROMs used to measure every PRO can be found in Additional file 5. For example, 77 different PROMs were used with the goal of measuring pain.

## Discussion

PROMs play an important role in incorporating the multidimensional health perspectives of children and their caregivers into daily care. By gaining a comprehensive understanding of the landscape of PROs and PROMs within PPT, along with identifying potentially valuable (generic) PROs for the specific context and population, the results of our study provide a foundation for developing recommendations for PROM use in PPT [23]. We identified 168 PROs which could be categorized into 40 unique PROs, measured with 158 different PROMs. Overall, most measured PROs were linked to the ‘Functional Status – Activities and Participation’ and ‘Symptom Status’ classification of the Alonso and Valderas model. This study provides a visual overview of PROs measured per medical diagnosis in PPT intervention studies, and therefore gives valuable information on these topics.

We encountered many variations in terminology for PROs, contributing to the large number of PROs found in the included studies of this scoping review. For example, considering the operationalization, the PROs ‘activities of daily living’, ‘activity’, ‘execution of a task or action’, and ‘activity performance’, could all be categorized within ‘general tasks and demands’ within the ICF. Previous studies also found many variations in PRO terminology. Terwee et al. (2021) identified common PROs in International Consortium for Health Outcomes Measurement (ICHOM) sets, and found 307 PROs which could be categorized into 22 unique PRO categories [19]. Another study condensing outcomes (both clinician and patient reported) used in clinical trials involving adults and children with genetic neurodevelopmental disorders and intellectual disability, found 438 reported outcomes which could be categorized into 91 unique outcomes [36]. Using different terminology for the same PRO complicates comparison and standardization of PRO data within and between diagnoses, leading to reduced clarity among intervention studies. Furthermore, heterogenic intervention strategies, such as home rehabilitation and multidisciplinary interventions, as well as the heterogenic population of children treated in PPT [27, 37], may contribute to the large number of PROs. However, our results show that overall, similar PROs are measured across all different medical diagnoses treated within PPT. This is in line with the overall emerging consensus within the field of PPT, where interventions focus on societal participation of children, with an emphasis on physical functioning, regardless of diagnosis [27, 29, 38, 39]. Previous studies also show overlapping PROs across different health conditions because of an overall desire of well-being and optimal functioning [19, 40]. A core set of generic PROs useful for the whole field of PPT could be beneficial. By using the same terminology, PRO data can be used and compared within and between diagnoses.

Furthermore, our results show that 158 different PROMs were used to measure 40 PROs and, on average, 17.4 PROMs were used to measure a single PRO, confirming the proliferation of PROMs. When considering the large amount of PROMs in combination with factors such as psychometric properties of the PROMs, the selection of appropriate PROMs for use in daily practice or scientific research can be difficult. For example, a systematic review on PROMs used for pediatric patients with sport related injuries identified 22 PROMs, of which just two were recommended for use based on the measured acceptability, feasibility, appropriateness, and psychometric properties [41]. Another study among pediatric physical therapists in acute care showed that participants saw the potential of PROM use, but did not know which PROMs were available or appropriate for their patient population, hampering implementation [12]. Within the field of pediatric healthcare, development of PROMs for different age groups and additional (parent) proxy PROMs could add to the proliferation of PROMs. Another reason may be that different research groups are working independently on PRO/PROM selection and development for specific patient populations. For example, within ICHOM standard sets, different PROMs are recommended to measure the same PRO for different patient groups [19]. This exemplifies another reason for the proliferation of PROMs, namely the use of disease-specific PROMs. Our study found that 41.8% of PROMs used in PPT are disease-specific. Although disease-specific measurements might contain relevant items for a specific population, there are several disadvantages in using them. Completing multiple different disease-specific PROMs (sometimes with overlapping PRO concepts) can be confusing for patients with multiple health conditions. Content analyses of disease-specific PROMs show that they mainly focus on a limited number of generic PROs [19, 33]. This may be because various diseases impact similar functions and symptoms, making the same generic PROs relevant across different diseases [19]. Overall, using numerous different PROMs (generic and disease specific), with different scoring systems, hampers the comparability and interpretation of outcomes across different diseases. As such, various expert workgroups stated the importance of using a core set of generic PROMs where possible, and to supplement them with disease-specific PROMs as needed [19, 21, 22]. For example, for patients with asthma treated in a hospital setting a core set of generic PRO(M)s (e.g. quality of life, fatigue and participation) could be supplemented with a disease-specific PROM about coughing, wheezing and shortness of breath, such as the PROMIS^®^ Asthma Impact Scale [42].

Our results show that goal attainment was frequently measured (Fig. 3). Outcome measures such as Goal Attainment Scaling (GAS) or the Canadian Occupational Performance Measure (COPM) were used. As goal attainment does not target a specific predetermined health domain, it qualifies as a PRO only when the goal relates to an outcome that can be directly reported by a patient. Regardless, measuring goal attainment aligns with the importance of personalized care in PPT [27, 43]. Goal attainment was particularly measured in studies involving children with chronic conditions. Children with chronic conditions often require long term goals with individualized intervention aims. Measuring goal attainment helps tailor the intervention to the child’s and caregiver’s needs [44–46]. Additionally, these measurements help study the effects of PPT interventions with heterogeneous aims, making research more clinically relevant by closely resembling daily practice [45].

This study shows that harmonization of the use of PRO and PROMs in PPT is indicated. To provide guidance on the selection and use of PROMs in PPT, we recommend the development of a core set of generic PROs and PROMs for PPT, which can be used in daily practice and research. This core set can be supplemented with disease-specific PROMs if needed. By using the same generic PROMs, data can be compared within and between diagnoses. Considering the framework *PROM Cycle*, supporting the selection and implementation of PROMs by Van der Wees et al. 2019 [47], the results of this review could be used for the selection of PROs for a PRO core set. It is important to involve all stakeholders of PPT interventions when defining a PRO core set, such as patients with different diagnoses, their caregivers, and pediatric physical therapists as well as researchers and PRO(M) experts to validate the results of our review. To gather support on an (inter)national level for a core set of PROs and PROMs, an expert panel should be involved in the operationalization and prioritization of PROs. Afterwards, matching PROMs with predetermined requirements, such as costs, usability and psychometric properties, can be selected for a PROM core set.

This review presents a comprehensive overview of the landscape of PROs and PROMs used in PPT, and provides the first systematic visual presentation of PROs measured across different diagnostic groups in PPT. This review serves as a starting point of harmonization of PRO(M) use within PPT. However, this research has some limitations. First, a large number of included studies (49 out of 172; 28.3%) reported on children with cerebral palsy, likely due to the increasing research on the condition [48]. This overrepresentation might have influenced the outcomes for the diagnostic group ‘chronic, neurological’. However, given the prevalence of children with cerebral palsy in PPT [37], the information remains highly relevant. Additionally, the inclusion of 29 studies on other chronic neurological diagnoses helps balancing the overrepresentation of cerebral palsy. Second, PROs are less measured in studies describing PPT interventions involving the diagnostic groups cardiovascular and respiratory (*n* = 9), oncology (*n* = 7), mental health (*n* = 4), and gastrointestinal, endocrine and hematological (*n* = 10), resulting in potential gaps of knowledge. However, we included some studies involving these diagnostic groups. Additionally, the same PROs are often relevant across different patient populations. Third, although the inclusion of 172 studies with a range of different diagnoses most likely gives a representative overview of measured PROs in PPT, the use of only one online database in our search might limit the results. Lastly, as the operationalizations of PROs were often unclear in the included studies, there is a possibility that PROs were not interpreted as intended by the authors. In general, we attempted to stay as close as possible to the authors’ intended operationalization, by reporting the PROs directly described in the paper, and only if absent, extracted them from the utilized PROMs. However, some nuance might be lost when categorizing the PROs.

## Conclusion

This review identified 168 PROs which could be categorized into 40 unique PROs, measured with 158 different PROMs. After categorizing the PROs, most fell in the ‘Functional Status – Activities and Participation’ and ‘Symptom Status’ classification of the Alonso and Valderas model. The excessive number of different PROs and PROMs used in PPT might complicate PROM implementation [12]. We recommend harmonizing and standardizing PROs and PROMs by developing a generic core set for use in daily practice and research in PPT internationally. The use of a core set would enhance comparability within and between diagnoses in PPT, and contribute to personalized interventions and improved patient-centered care. This review may serve as a starting point for identifying a generic core set of PROs for PPT.

## Supplementary Information

Below is the link to the electronic supplementary material.

Supplementary Material 1

Supplementary Material 2

Supplementary Material 3

Supplementary Material 4

Supplementary Material 5

## Data Availability

The datasets used and/or analyzed during the current study are available from the corresponding author on reasonable request.

## Abbreviations

COPM: Canadian Occupational Performance Measure
GAS: Goal Attainment Scaling
ICF: International Classification of Functioning, Disability and Health
ICHOM: International Consortium for Health Outcomes Measurement
PedsQL 4.0: Pediatric Quality of Life Inventory
PPT: Pediatric physical therapy
PRISMA-ScR: Preferred Reporting Items for Systematic reviews and Meta-Analysesextension for Scoping Reviews
PRO: Patient-reported outcome
PROM: patient-reported outcome measure
PROMIS®: Patient-Reported Outcomes Measurement Information System^®^
VOS: Visualization of similarities
