# Supplementary material for: Patient reported outcomes in pediatric physical therapy: a scoping review and evidence map
Source: J Patient Rep Outcomes. 2025 Oct 24;9:125. doi: 10.1186/s41687-025-00947-5 (PMC12552199; doi:10.1186/s41687-025-00947-5)
Supplement: Supplementary file 1 — Supplementary Material 1 [file 41687_2025_947_MOESM1_ESM.docx]

**Additional file 1: Search strategy**

| **Ovid MEDLINE(R) ALL <1946 to October 05, 2023> Search date: 6 October 2023** | | |
| --- | --- | --- |
| **#** | **Searches** | **Results** |
| 1 | (physiotherap* or exercise therap* or (physical adj3 (intervention? or therap* or exercis*)) or hand therap*).mp. | 169595 |
| 2 | ((fitness or physical activit* or exercis*) and rehabilitation).mp. | 55346 |
| 3 | (rehabilitation adj (service? or outpatient? or inpatient? or unit?)).mp. | 9489 |
| 4 | (rehabilitation adj3 (child* or pediatric or youth? or boy or boys or girl?)).mp. | 3813 |
| 5 | ("return to sports" or "return to play").mp. | 5224 |
| 6 | ergotherap*.mp. | 544 |
| 7 | occupational therap*.mp. | 24108 |
| 8 | or/1-7 [I] | 226784 |
| 9 | exp child/ or adolescent/ or exp child welfare/ | 3384210 |
| 10 | (adolescen* or kid or kids or underage* or boy or boys or girl? or sibbling* or preschool* or childhood or child or children or schoolchild* or juvenile or minors or p?ediatric? or school age).ab,kf,ti. | 2210522 |
| 11 | (("4" or "5" or "6" or "7" or "8" or "9" or "10" or "11" or "12" or "13" or "14" or "15" or "16" or "17" or "18") adj1 (age? or yr? or year?)).ab. | 1477708 |
| 12 | (child or p?ediatric? or juvenile).jw. | 654149 |
| 13 | or/9-12 [II children 4 - 18 yrs] | 4972297 |
| 14 | exp Patient Outcome Assessment/ or self report/ | 65026 |
| 15 | patient reported outcome?.ab,kf,ti. | 38213 |
| 16 | self report*.ab,kf,ti. | 209583 |
| 17 | or/14-16 | 265888 |
| 18 | (Mental or Emotional or Anxiety or Depression or depressive or Anger or (Physical* adj2 function*) or Physical activit* or Upper extremit* or lower extremit* or Mobility or ((sport? or physical or Social) adj2 (function* or participation)) or Family or Leisure time or School or (life adj3 quality) or qol or Pain or ((percept* or Perceived) adj2 health) or Sleep or Fatigue or (cognitive adj2 (function* or dysfunction*)) or Sexuality or Self-confidence or esteem or Resilience or Coping or Self-efficacy).mp. | 4561211 |
| 19 | ((child* or parent? or caregiver? or family or proxy or self or patient?) adj3 (report* or experience? or perceive?)).mp. | 781699 |
| 20 | 18 and 19 | 373775 |
| 21 | ((Mental or Emotional or Anxiety or Depression or depressive or Anger or (Physical* adj2 function*) or Physical activit* or Upper extremit* or lower extremit* or Mobility or ((sport? or physical or Social) adj2 (function* or participation)) or Family or Leisure time or School or (life adj3 quality) or qol or Pain or ((percept* or Perceived) adj2 health) or Sleep or Fatigue or (cognitive adj2 (function* or dysfunction*)) or Sexuality or Self-confidence or esteem or Resilience or Coping or Self-efficacy) adj3 (experience or perceive?)).mp. | 72456 |
| 22 | ("Abilhand-Kids" or "Activities-specific Balance Confidence Scale" or "Activity Scale for Kids" or "ADHD-vragenlijst" or "Adolescent / Adult Sensory Profile" or "Adolescent Sleep Hygiene Questionnaire" or "Adolescent Sleep Hygiene Scale" or "Ages & Stages Questionnaires" or "Amsterdam Sexual Pleasure Index" or "Assessment of Preschool Children's Participation" or "Assessment of Quality of Life" or "Asthma Control Questionnaire" or "Asthma Control Test" or "Attachment Insecurity Screening Inventory" or "Baecke" or "Beck Anxiety Inventory" or "Beck Depression Inventory" or "Behavior Assessment System for Children" or "Behavior Rating Inventory of Executive Function" or "Behavioral Pediatrics Feeding Assessment Scale" or "Beliefs about Medicine Questionnaire" or "Benefit and Burden Scale for Children" or "Brief Calling Scale" or "Brief infant sleep Q" or "Brief ouder executive functies" or "Brief Problem Monitor" or "Brief Symptom Inventory" or "Bristol Stoelgang[1]schaal" or "Canadian occupational performance measure" or "Cantril Ladder of Life" or "Care-related Quality of Life instrument" or "Center for Epidemiologic Studies Depression scale" or "Checklist Individual Strength" or "Checklist individuele spankracht" or "Child and Adolescent Needs and Strengths" or "Child and Adolescent Scale of Participation" or "Child Behavior Checklist" or "Child Health Questionnaire" or "Child Oral Health Impact Profile for children with orofacial anomalies" or "Child Perception Questionnaire" or "Child Rating Scale" or "Child Sleep Hygiene Questionnaire" or "Child Sleep Hygiene Scale" or "Child Status Index" or "Childhood Asthma Control Test" or "CHILDHOOD HEALTH ASSESSMENT QUESTIONNAIRE" or "Children's assessment of participation and enjoyment & preferences for activities of children" or "Children's Self-Perception of Adequacy in and Predi lection toward Physical Activity" or "Children's Behavior Questionnaire" or "Children's Depression Inventory" or "Children's Dermatology Life Quality Index" or "Children's Dermatology Life Quality Index" or "Children's Hand-use Experience Questionnaire" or "Children's Revised Impact of Event Scale" or "Children's Wellbeing at School" or "Children's Yale-Brown Obsessive Compulsive Scale" or "Chronic Sleep Reduction Questionnaire" or "Cleft Questionnaire " or "Cognitive Emotion Regulation Questionnaire" or "Cognitive Functioning Self-Assessment Scale" or "Competentie belevingsschaal voor kinderen" or "Competentie-Belevingsschaal voor Adolescenten" or "Competentiebelevingsschaal voor Kinderen" or "Coördinatievragenlijst Voor Ouders" or "Coördinatievragenlijst voor Ouders" or "coping health inventory for parents " or "Coping Strategies Questionnaire" or "Dagelijkse participatie - Q-koortsvermoeidheidssyndroom" or "DCDDaily-Q" or "Dental Discomfort Questionnaire" or "Depression, Anxiety and Stress Scale" or "Developmental Screening Inventory" or "DISABKIDS" or "DUTCH-Children-AZL TNO" or "Early Screening of Autistic Traits" or "European Organization for Research and Treatment of Cancer Quality-of-Life questionnaire cervical cancer module" or "EuroQol" or "Faces Pain Scale – Revised" or "Family Adaptability and Cohesion EvaluationScalesII" or "Family Assessment Device" or "Family Environment Scale" or "Fatigue Questionnaire" or "FEEL-KJ" or "Female Sexual Distress Scale-Revised" or "Female Sexual Function Index" or "Functional Disability Inventory" or "Functional Status II" or "Functional Status Questionnaire II" or "Goal Attainment Scale" or "Gedrags-vragenlijst" or "Gedragsvragenlijst voor Kleuters" or "General Anxiety Disorder-7" or "Gezinsvragenlijst" or "Gezond-heidsbeleving" or "Global Assessment of Functioning" or "Groeiwijzer" or "Groninger Motoriek Observatie schaal" or "Happy inflammatory bowel disease" or "Health Utilities Index" or "Health-of-the-Nation-Outcome-Scales Children " or "Hoe vind ik dat ik het doe" or "Hospital Anxiety and Depression Scale" or "Hospital for Special Surgery Pediatric Functional Activity Brief Scale" or "Illness Perception Questionnaire" or "Impact of Weight on Quality of Life" or "IMPACT-III" or "Injury Severity Score" or "Insomnia severity index" or "INVENTORY OF DEPRESSIVE SYMPTOMATOLOGY" or "Junior Nederlandse Persoonlijkheidsvragenlijst" or "Junior Nederlandse Persoonlijkheidsvragenlijst 2" or "KidCope" or "Kidscreen" or "KINDL(-R)" or "KNO-Otologic Health" or "KOOS-Child" or "Last Thermometer voor Ouders" or "Lastmeter PSZ" or "Lexilijst A" or "Lexilijst B" or "Life engagement scale" or "Life Events Checklist" or "Life-H" or "lifestyle assessment questionnaire for children" or "Modifiable Activity Questionnaire" or "Multidimensional Fatigue Inventory" or "Multidimensional Scale of Perceived Social Support" or "Nederlandse Vragenlijst voor Eetgedrag" or "NIH Toolbox Self-Efficacy CAT" or "nijmeegse ouderlijke stress index" or "Numeric Rating Scale" or "Nutrition in Pediatric Critical Care" or "obstructive sleep apnea" or "Opvoedingsbelasting Vragenlijst" or "Ouder-Kind Interactie Vragenlijst-Revised" or "OVAMA" or "Pain Catastrophizing Scale" or "parent-reported pediatric perceived cognitive functioning" or "Participation and Environment Measure for Children and Youth" or "Patient Health Questionnaire" or "Patient Perception of Patient-Centeredness " or "Pediatric Asthma Caregiver's Quality of Life Questionnaire" or "Pediatric Asthma Quality of Life Questionnaire" or "Pediatric Evaluation of Disability Inventory" or "Pediatric Quality of Life Inventory" or "Pediatric Quality of Life Inventory Fatique " or "Penn State Worry Questionnaire" or "Perceived Efficacy and Goal Setting System" or "Perceived Stigmatisation Questionnaire" or "Perceived Stress Questionnaire" or "Perceived Stress Scale" or "Person-Centred Coordinated Care Experience Questionnaire" or "Physical Activity Readiness Questionnaire" or "Positieve gezondheid" or "Probleemvragen ASR" or "Problem Areas in Diabetes Questionnaire" or "PROMIS Asthma Impact" or "PROMIS Cognitive Function" or "PROMIS Emotional Distress – Anger" or "PROMIS Emotional Distress – Anxiety" or "PROMIS Emotional Distress – Depressive Symptoms" or "PROMIS Family Relationships" or "PROMIS Fatigue" or "PROMIS Global Health" or "PROMIS Life Satisfaction" or "PROMIS Meaning and Purpose" or "PROMIS Pain – Behavior" or "PROMIS Pain – Interference" or "PROMIS Pediatric/Parent Proxy Profile 25" or "PROMIS Pediatric/Parent Proxy Profile 37" or "PROMIS Pediatric/Parent Proxy Profile 49" or "PROMIS Peer Relationships" or "PROMIS Physical Activity" or "PROMIS Physical Function – Mobility" or "PROMIS Physical Function – Upper Extremity" or "PROMIS Physical Stress Experience" or "PROMIS Sleep Disturbance" or "PROMIS Sleep-Related Impairment " or "PROMIS Strength Impact" or "Psychosocial Assessment Tool" or "PTSS Checklist" or "Rand General Health Rating Index for Children" or "Recap of atopic eczema" or "Resilience Evaluation Scale" or "Revised Children's Anxiety and Depression Scale" or "Roland‐Morris Disability Questionnaire" or "RSE Zelfwaardering" or "Satisfaction With Life Scale" or "Satisfaction with life scale for children (Diener's)" or "SCARED" or "Schokverwerkingslijst voor Kinderen" or "School NF" or "School Questionnaire For Teachers" or "Self Efficacy Scale" or "Self-Administered Eczema Area and Severity Index" or "Self-Efficacy Questionnaire for Children" or "Self-Efficacy Vragenlijst - QVS" or "Self-Perception Profile for Children" or "Sensory Profile-NL" or "Short-Form Health Survey" or "SKINDEX-29" or "Sleep Disturbance Scale for Children" or "sleep self report" or "slow-wave activity" or "Social Anxiety Scale for Adolescents" or "Social Communication Questionnaire" or "Social Phobia and Anxiety Inventory for Children" or "Social Responsiveness Scale" or "State-Trait Anxiety Inventory for Children" or "Strengths and Difficulties Questionnaire" or "Strengths and Weaknesses of Attention-Deficit/Hyperactivity Disorder Symptoms and Normal Behavior Scale" or "Survey Highly Sensitive Children" or "Symptom Checklist-90 " or "Teacher's Report Form" or "The Children's Communication Checklist" or "The Genderqueer Identity Scale" or "TNO AZL Child Quality of Life" or "Utrecht Scale for Evaluation of Rehabilitation-Participation" or "Verbetering Vragenlijst voor tieners en jongvolwassenen - QVS" or "Vineland screener / hogrefe" or "Vineland Z" or "Visual analog scale astma" or "Vragenlijst Lichamelijke Klachten-Kinderen" or "Vragenlijst over Ontwikkeling en Gedrag" or "Vragenlijst voor Gedragsproblemen bij Kinderen" or "Vragenlijst voor Inventarisatie van Sociaal gedrag van Kinderen" or "Weiss Functional Impairment Rating Scale" or "WHO Disability Assessment Schedule 12" or "World Health Organization Qualitiy of Life Questionnaire" or "YCND Baecke Vragenlijst" or "Youth Quality of Life Instrument" or "Youth Self Report" or "Zelfinventarisatielijst Posttraumatische Stressstoornis" or "Ziektelastmeter").mp. | 167220 |
| 23 | ("AVL" or "AASP-NL" or "ASHQ" or "ASHS" or "ASQ" or "ASPI" or "APCP" or "AQOL-8D" or "ACQ" or "ACT" or "AISI" or "BAI" or "BRIEF" or "BPFAS" or "BMQ" or "BBSC" or "BCS" or "BPM" or "BSI" or "COPM" or "CLL" or "CarerQol V1" or "CES-D" or "CIS" or "CANS-MH" or "CASP" or "CBCL" or "CHQ" or "COHIP OSS" or "CRS" or "CSHQ" or "CSHS" or "CSI" or "C-ACT" or "CHAQ" or "CAPE/PAC" or "CSAPPA" or "CBQ" or "CDI en CDI-2 31" or "CDLQI" or "DLQI" or "CHEQ" or "CRIES" or "QWBS" or "CY-BOCS" or "CSRQ" or "CLEFT-Q" or "CERQ" or "CFSS" or "CBSK" or "CBSA" or "CBSK" or "CVO" or "CVO" or "CHIP" or "CSQ" or "Dagelijkse participatie – QVS" or "DDQ 0-5" or "DASS-21" or "DSI" or "DUCATQOL" or "ESAT" or "EORTC QLQ CX24" or "eq 5d" or eq5 or "eq 5 d" or "FPS-R" or "FAD" or "CIS 20" or "FSDS-R" or "FSFI" or "FDI" or "FS-II" or "GAS" or "GvK" or "GAD-7" or "GVL" or "GAF" or "GMO" or "Happy IBD" or "HUI" or "HoNOS Child" or "HADS" or "HSS Pedi-FABS" or "Self IPQ-K" or "IWQOL" or "ISS" or "IDS" or "NPV(J)-2" or "LTO" or "LEC-5" or "LAQ-CP" or "MAQ" or "MFI" or "MSPSS" or "NVE" or "NOSIK" or "NRS" or "OSA-12" or "OBVL" or "OKIV-R" or "PCS-C" or "PedsPCF" or "PEM-CY" or "PHQ-9" or "PPPC-R" or "PACQLQ" or "PAQLQ" or "PEDI" or "PedsQL" or "PedsQL Fatigue" or "PSWQ" or "PEGS" or "PSQ" or "PSS" or "PAR-Q" or "PAID-20" or "PAT" or "PCL-5" or "RAND" or "RECAP" or "RCADS" or "RMQ" or "SWLS/SHS" or "SVLK" or "SQT" or "SES" or "SA-EASI" or "SCSES" or "SP-NL" or "SF-12" or "SDSC" or "SWA" or "SAS-A" or "SCQ" or "SPAI-C" or "SRS-2" or "STAI-STATE" or "SDQ" or "SWAN" or "HSC" or "SCL-90-R" or "C-TRF" or "CCC-2" or "GQI Scale" or "TAPQOL" or "USER-P" or "VLK-K" or "VOG" or "VvGK" or "VISK" or "WFIRS" or "WHOQOL-Bref" or "YSR" or "ZIL-PTSS" or "CAPE" or "CBSK-M" or "DCD-Questionnaire" or "DCDQ" or "DUX25" or "PEDI-CAT" or "PEDI-NL" or "PEDI-IKDC").mp. | 1432776 |
| 24 | 22 or 23 [measurement tools] | 1542420 |
| 25 | or/17,20-21,24 | 1994485 |
| 26 | and/8,13,25 | 7917 |
| 27 | (Vegetable or Fruit or Self determination theory or Control school or Covid or Pandamic or Lockdown or Coronavirus disease or Employee or Outbreak or Alcohol or Alcohol consumption or (Consumer not consumer driven) or Sars cov or Smoking or Obese adult or Television or Goverment or Fidelity or Cochlear implant or Law or Office or (Breast adj2 (cancer or neoplasm?)) or nursing or robot or total knee or platelet rich plasma or college or curricul*).ab,ti. [VOS NOTing out] | 2217523 |
| 28 | exp animals/ not humans/ | 5160847 |
| 29 | 26 not (27 or 28) | 6906 |
| 30 | limit 29 to yr="2013-current" | 4614 |
|  |  |  |
