# Supplementary material for: Patient reported outcomes in pediatric physical therapy: a scoping review and evidence map
Source: J Patient Rep Outcomes. 2025 Oct 24;9:125. doi: 10.1186/s41687-025-00947-5 (PMC12552199; doi:10.1186/s41687-025-00947-5)
Supplement: Supplementary file 2 — Supplementary Material 2 [file 41687_2025_947_MOESM2_ESM.docx]

**Additional file 2: The Alonso & Valderas model including adaptations for this study**

To provide a structured overview of PROs within a graphical format, PRO terminologies were labelled according to the Alonso & Valderas model [1], a classification system of PRO measures. The Alonso & Valderas model combines the ICF and the Wilson and Cleary model [2], a bio-psycho-social model for health outcomes. To create more nuances within the classification ‘Functional Status’ of the Alonso & Valderas model, sub-categories related to an individual’s functioning were incorporated based on the PROMIS® conceptual framework, in alignment with research performed by Oude Voshaar et al. [3]. The additional subcategories were ‘Social Health’ and ‘Mental Health’. As a result, the ‘Functional Status’ domain now comprises the following subcategories: ‘Activities and Participation’, ‘Social Health’ and ‘Mental Health’. See Figure 1.


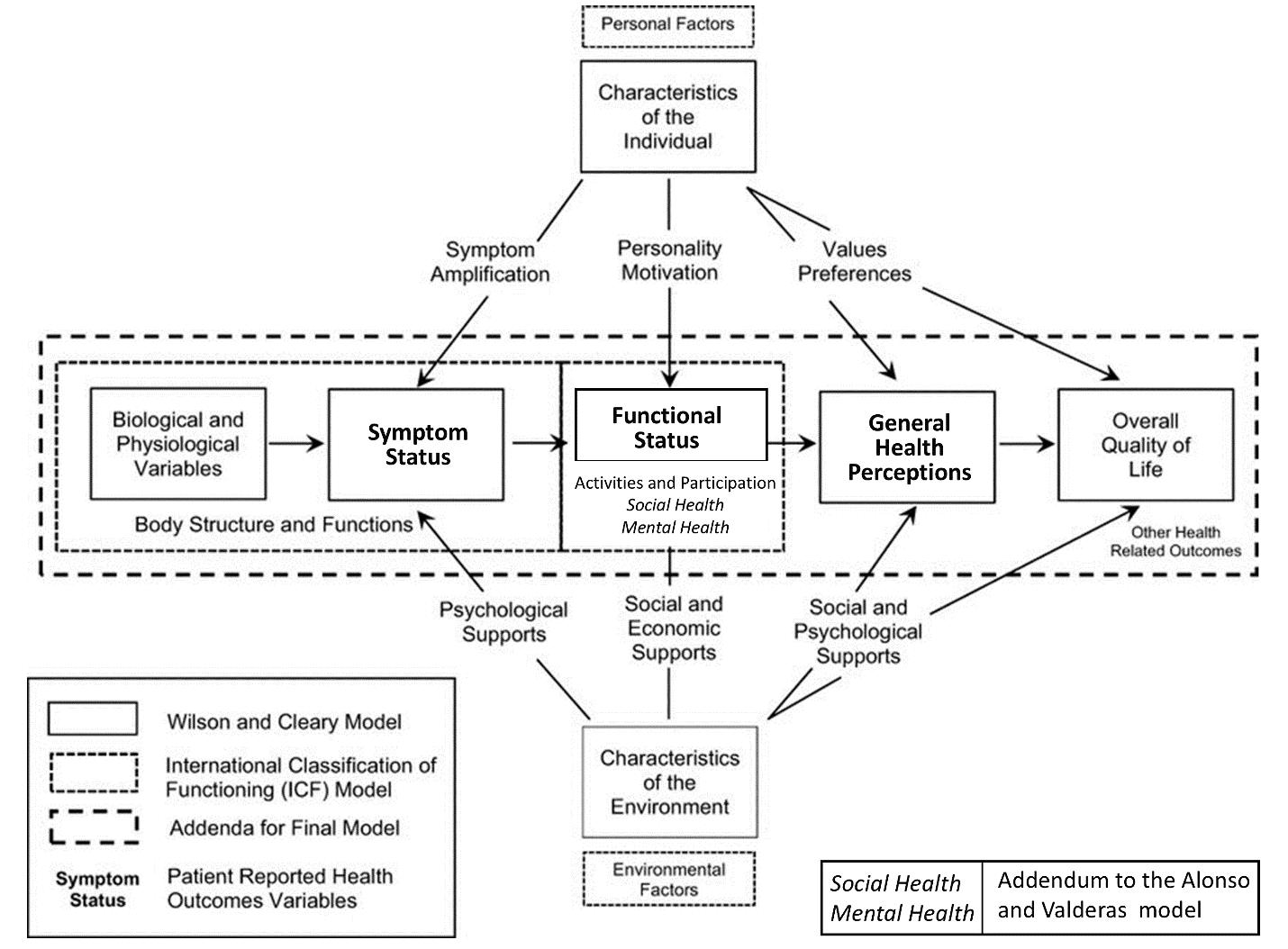


Figure 1: The Alonso and Valderas model, with two additional sub-categories within the ‘Functional Status’ category. The additional sub-categories were added based on the Patient-Reported Outcomes Measurement Information System® (PROMIS®) conceptual framework [31]. Adapted from Alonso and Valderas [33].
