# Supplementary material for: Patient reported outcomes in pediatric physical therapy: a scoping review and evidence map
Source: J Patient Rep Outcomes. 2025 Oct 24;9:125. doi: 10.1186/s41687-025-00947-5 (PMC12552199; doi:10.1186/s41687-025-00947-5)
Supplement: Supplementary file 3 — Supplementary Material 3 [file 41687_2025_947_MOESM3_ESM.docx]

**Additional file 3: References of the included studies**

| 1. Abd-Elmonem AM, Al-Tohamy AM, Galal RE, Abd-Elhalim FA. Effects of progressive resistance exercises on quality of life and functional capacity in pediatric patients with chronic kidney disease: a randomized trail. J Musculoskelet Neuronal Interact. 2019;19(2):187-195. |
| --- |
| 1. Abdelbasset WK, Elsayed SH, Nambi G, et al. Optimization of pulmonary function, functional capacity, and quality of life in adolescents with thoracic burns after a 2-month arm cycling exercise programme: A randomized controlled study. Burns : journal of the International Society for Burn Injuries. 2022;48(1):78-84. doi:10.1016/j.burns.2021.03.010 |
| 1. Adler C, Hessenauer M, Lipp J, et al. Learning to cope with mirror movements in unilateral spastic cerebral palsy: a brief report. Dev Neurorehabil. 2019;22(2):141-146. doi:10.1080/17518423.2018.1474501 |
| 1. Allonsius F, De Kloet AJ, Van Markus-Doornbosch F, Meesters JJL, Kromme CH, Vliet Vlieland TPM, Van Der Holst M. Parent-reported family impact in children and young adults with acquired brain injury in the outpatient rehabilitation setting. Brain Inj. 2021;35(5):563-573. doi:10.1080/02699052.2021.1891287 |
| 1. Alphazab AR, Elnaggar RK, Diab RH, Moawd SA. Therapeutic value of kinesio taping in reducing lower back pain and improving back muscle endurance in adolescents with hemophilia. J Musculoskelet Neuronal Interact. 2020;20(2):256-264. |
| 1. An M, Palisano RJ, Yi C-H, Chiarello LA, Dunst CJ, Gracely EJ. Effects of a Collaborative Intervention Process on Parent Empowerment and Child Performance: A Randomized Controlled Trial. Phys Occup Ther Pediatr. 2019;39(1):1-15. doi:10.1080/01942638.2017.1365324 |
| 1. Araneda R, Herman E, Delcour L, et al. Mirror movements after bimanual intensive therapy in children with unilateral cerebral palsy: A randomized controlled trial. Dev Med Child Neurol. 2022;64(11):1383-1391. doi:10.1111/dmcn.15257 |
| 1. Arman N, Tarakci E, Tarakci D, Kasapcopur O. Effects of Video Games-Based Task-Oriented Activity Training (Xbox 360 Kinect) on Activity Performance and Participation in Patients With Juvenile Idiopathic Arthritis: A Randomized Clinical Trial. Am J Phys Med Rehabil. 2019;98(3):174-181. doi:10.1097/PHM.0000000000001001 |
| 1. Armstrong EL, Boyd RN, Horan SA, Kentish MJ, Ware RS, Carty CP. Functional electrical stimulation cycling, goal-directed training, and adapted cycling for children with cerebral palsy: a randomized controlled trial. Dev Med Child Neurol. 2020;62(12):1406-1413. doi:10.1111/dmcn.14648 |
| 1. Arnevik Austrheim K, Skagen C, Rieber J, Melfald Tveten K. Practice, play, repeat - individualized outcomes after the "intensity matters!"-program for children with disabilities - a descriptive multicase study. Disabil Rehabil. 2023:1-6. doi:10.1080/09638288.2023.2233905 |
| 1. Arrebola LS, Yi LC, de Oliveira VGC. The use of video games combined with conventional physical therapy in children with upper limb fractures: An exploratory study. J Pediatr Rehabil Med. 2019;12(1):65-70. doi:10.3233/PRM-170529 |
| 1. Ashkenazi T, Weiss PL, Orian D, Laufer Y. Low-cost virtual reality intervention program for children with developmental coordination disorder: a pilot feasibility study. Pediatr Phys Ther. 2013;25(4):467-73. doi:10.1097/PEP.0b013e3182a74398 |
| 1. Atasavun Uysal S, Baltaci G. Effects of Nintendo Wii TM Training on Occupational Performance, Balance, and Daily Living Activities in Children with Spastic Hemiplegic Cerebral Palsy: A Single-Blind and Randomized Trial. Games Health J. 2016;5(5):311-317. doi:10.1089/g4h.2015.0102 |
| 1. Atkinson M, Tully A, Maher CA, Innes-Wong C, Russo RN, Osborn MP. Safety, Feasibility and Efficacy of Lokomat R and Armeo RSpring Training in Deconditioned Paediatric, Adolescent and Young Adult Cancer Patients. Cancers. 2023;15(4)doi:10.3390/cancers15041250 |
| 1. Aubry-Rozier B, Richard C, Unger S, et al. Osteogenesis imperfecta: towards an individualised interdisciplinary care strategy to improve physical activity and quality of life. Swiss Med Wkly. 2020;150:w20285. doi:10.4414/smw.2020.20285 |
| 1. Aukstikalnis T, Sinkevicius R, Rasimaite O, et al. The Effect of comprehensive rehabilitation on Lithuanian adolescent's nonspecific low back pain, depending on the duration: Nonrandomized single-arm trial. Medicine. 2022;101(41):e30940. doi:10.1097/MD.0000000000030940 |
| 1. Bae DS, Shah AS, Kalish LA, Kwon JY, Waters PM. Shoulder motion, strength, and functional outcomes in children with established malunion of the clavicle. J Pediatr Orthop. 2013;33(5):544-50. doi:10.1097/BPO.0b013e3182857d9e |
| 1. Bailey C, Meyer J, Briskin S, et al. Multidisciplinary Concussion Management: A Model for Outpatient Concussion Management in the Acute and Post-Acute Settings. J Head Trauma Rehabil. 2019;34(6):375-384. doi:10.1097/HTR.0000000000000527 |
| 1. Baksjoberget PE, Nyquist A, Moser T, Jahnsen R. Having Fun and Staying Active! Children with Disabilities and Participation in Physical Activity: A Follow-Up Study. Phys Occup Ther Pediatr. 2017;37(4):347-358. doi:10.1080/01942638.2017.1281369 |
| 1. Barak S, Silberg T, Gerner M, Eisenstein E, Bardach CA, Yissar T, Landa J. Functional neurological symptom disorder: Preliminary findings of factors associated with walking ability post integrative pediatric rehabilitation. J Pediatr Rehabil Med. 2022;15(3):433-446. doi:10.3233/PRM-210107 |
| 1. Barnes E, Hillier-Moses G, Murray H, Stevinson C, Franks HA, Gossage L. Evaluation of the MOVE online exercise programme for young people aged 13-30. Support Care Cancer. 2023;31(7):377. doi:10.1007/s00520-023-07758-8 |
| 1. Baydogan SN, Tarakci E, Kasapcopur O. Effect of strengthening versus balance-proprioceptive exercises on lower extremity function in patients with juvenile idiopathic arthritis: a randomized, single-blind clinical trial. Am J Phys Med Rehabil. 2015;94(6):417-8. doi:10.1097/PHM.0000000000000279 |
| 1. Benore E, D'Auria A, Banez GA, Worley S, Tang A. The influence of anxiety reduction on clinical response to pediatric chronic pain rehabilitation. Clin J Pain. 2015;31(5):375-83. doi:10.1097/AJP.0000000000000127 |
| 1. Bernstein R, Getzoff E, Gelfand K, Demeule-Hayes M, Scheimann A. Interaction and influence of child and family characteristics upon success of weight management treatment. Eat Weight Disord. 2021;26(6):2033-2041. doi:10.1007/s40519-020-01052-w |
| 1. Bingol H, Gunel MK. Comparing the effects of modified constraint-induced movement therapy and bimanual training in children with hemiplegic cerebral palsy mainstreamed in regular school: A randomized controlled study. Arch Pediatr. 2022;29(2):105-115. doi:10.1016/j.arcped.2021.11.017 |
| 1. Blanco Diaz M, Bousono Garcia C, Segura Ramirez DK, Rodriguez Rodriguez AM. Manual Physical Therapy in the Treatment of Functional Constipation in Children: A Pilot Randomized Controlled Trial. J Altern Complement Med. 2020;26(7):620-627. doi:10.1089/acm.2020.0047 |
| 1. Bleyenheuft Y, Arnould C, Brandao MB, Bleyenheuft C, Gordon AM. Hand and Arm Bimanual Intensive Therapy Including Lower Extremity (HABIT-ILE) in Children With Unilateral Spastic Cerebral Palsy: A Randomized Trial. Neurorehabil Neural Repair. 2015;29(7):645-57. doi:10.1177/1545968314562109 |
| 1. Bleyenheuft Y, Ebner-Karestinos D, Surana B, et al. Intensive upper- and lower-extremity training for children with bilateral cerebral palsy: a quasi-randomized trial. Dev Med Child Neurol. 2017;59(6):625-633. doi:10.1111/dmcn.13379 |
| 1. Boucher B, Smith-Young B. Examination and physical therapy management of a young gymnast with bilateral wrist pain: A case report. Phys Ther Sport. 2017;27:38-49. doi:10.1016/j.ptsp.2017.03.002 |
| 1. Brandao MB, Mancini MC, Ferre CL, et al. Does Dosage Matter? A Pilot Study of Hand-Arm Bimanual Intensive Training (HABIT) Dose and Dosing Schedule in Children with Unilateral Cerebral Palsy. Phys Occup Ther Pediatr. 2018;38(3):227-242. doi:10.1080/01942638.2017.1407014 |
| 1. Brandao MB, Oliveira RHS, Mancini MC. Functional priorities reported by parents of children with cerebral palsy: contribution to the pediatric rehabilitation process. Braz J Phys Ther. 2014;18(6):563-71. doi:10.1590/bjpt-rbf.2014.0064 |
| 1. Bruce BK, Harrison TE, Bee SM, et al. Improvement in Functioning and Psychological Distress in Adolescents With Postural Orthostatic Tachycardia Syndrome Following Interdisciplinary Treatment. Clin Pediatr (Phila). 2016;55(14):1300-1304. doi:10.1177/0009922816638663 |
| 1. Bruce BK, Weiss KE, Ale CM, Harrison TE, Fischer PR. Development of an Interdisciplinary Pediatric Pain Rehabilitation Program: The First 1000 Consecutive Patients. Mayo Clin Proc Innov Qual Outcomes. 2017;1(2):141-149. doi:10.1016/j.mayocpiqo.2017.05.006 |
| 1. Butts R, Legaspi O, Nocera-Mekel A, Dunning J. Physical therapy treatment of a pediatric patient with symptoms consistent with a spinal cord injury without radiographic abnormality: A retrospective case report. J Bodyw Mov Ther. 2021;27:455-463. doi:10.1016/j.jbmt.2021.01.008 |
| 1. Cain MS, Ban RJ, Chen Y-P, Geil MD, Goerger BM, Linens SW. Four-Week Ankle-Rehabilitation Programs in Adolescent Athletes With Chronic Ankle Instability. J Athl Train. 2020;55(8):801-810. doi:10.4085/1062-6050-41-19 |
| 1. Cardenas A, Warner D, Switzer L, Graham TCN, Cimolino G, Fehlings D. Inpatient Exergames for Children with Cerebral Palsy following Lower Extremity Orthopedic Surgery: A Feasibility Study. Dev Neurorehabil. 2021;24(4):230-236. doi:10.1080/17518423.2020.1858359 |
| 1. Choi EJ, Kim W, Jeon JY, et al. Intensive pulmonary rehabilitation in a pediatric lung transplantation patient: A case report. Medicine. 2021;100(17):e25523. doi:10.1097/MD.0000000000025523 |
| 1. Choubisa CA, Jain M, Vardhan V, Budhwani YJ, Hege AR. Concomitant Pre- and Post-splenectomy Physiotherapy Rehabilitation in a 17-Year-Old Patient With Beta Thalassemia Major: A Case Report. Cureus. 2022;14(10):e29999. doi:10.7759/cureus.29999 |
| 1. Clutterbuck GL, Auld ML, Johnston LM. SPORTS STARS: a practitioner-led, peer-group sports intervention for ambulant children with cerebral palsy. Activity and participation outcomes of a randomised controlled trial. Disabil Rehabil. 2022;44(6):948-956. doi:10.1080/09638288.2020.1783376 |
| 1. Cohen-Holzer M, Sorek G, Kerem J, Katz-Leurer M. The impact of combined constraint-induced and bimanual arm training program on the perceived hand-use experience of children with unilateral cerebral palsy. Dev Neurorehabil. 2017;20(6):355-360. doi:10.1080/17518423.2016.1238017 |
| 1. Connell T, Paterson J, Roberts RM, Raghavendra P, Sawyer M, Russo RN. Clinician modifiable factors associated with better quality of life in children with acquired brain injury undergoing rehabilitation. Brain Inj. 2018;32(4):423-430. doi:10.1080/02699052.2018.1429661 |
| 1. Coronado RA, Sterling EK, Fenster DE, et al. Cognitive-behavioral-based physical therapy to enhance return to sport after anterior cruciate ligament reconstruction: An open pilot study. Phys Ther Sport. 2020;42:82-90. doi:10.1016/j.ptsp.2020.01.004 |
| 1. Curtis DJ, Woollacott M, Bencke J, Lauridsen HB, Saavedra S, Bandholm T, Sonne-Holm S. The functional effect of segmental trunk and head control training in moderate-to-severe cerebral palsy: A randomized controlled trial. Dev Neurorehabil. 2018;21(2):91-100. doi:10.1080/17518423.2016.1265603 |
| 1. David MMC, Gomes ELdFD, Mello MC, Costa D. Noninvasive ventilation and respiratory physical therapy reduce exercise-induced bronchospasm and pulmonary inflammation in children with asthma: randomized clinical trial. Ther Adv Respir Dis. 2018;12:1753466618777723. doi:10.1177/1753466618777723 |
| 1. Decavele S, Ortibus E, Van Campenhout A, Molenaers G, Jansen B, Omelina L, Franki I. The Effect of a Rehabilitation Specific Gaming Software Platform to Achieve Individual Physiotherapy Goals in Children with Severe Spastic Cerebral Palsy: A Randomized Crossover Trial. Games Health J. 2020;doi:10.1089/g4h.2019.0097 |
| 1. Delafontaine A, Presedo A, Mohamed D, Lopes D, Wood C, Alberti C. Equimolar mixture of nitroux oxyde and oxygen during post-operative physiotherapy in patients with cerebral palsy: A randomized, double-blind, placebo-controlled study. Eur J Pain. 2017;21(10):1657-1667. doi:10.1002/ejp.1071 |
| 1. Della Villa F, Straub RK, Mandelbaum B, Powers CM. Confidence to Return to Play After Anterior Cruciate Ligament Reconstruction Is Influenced by Quadriceps Strength Symmetry and Injury Mechanism. Sports health. 2021;13(3):304-309. doi:10.1177/1941738120976377 |
| 1. Dimitrova R, Kim H, Meilahn J, et al. Efficacy and safety of onabotulinumtoxinA with standardized physiotherapy for the treatment of pediatric lower limb spasticity: A randomized, placebo-controlled, phase III clinical trial. NeuroRehabilitation. 2022;50(1):33-46. doi:10.3233/NRE-210070 |
| 1. Dobney DM, Grilli L, Kocilowicz H, Beaulieu C, Straub M, Friedman D, Gagnon IJ. Is There an Optimal Time to Initiate an Active Rehabilitation Protocol for Concussion Management in Children? A Case Series. J Head Trauma Rehabil. 2018;33(3):E11-E17. doi:10.1097/HTR.0000000000000339 |
| 1. Dodds CB, Bjornson KF, Sweeney JK, Narayanan UG. The effect of supported physical activity on parental-reported health-related quality of life in children with medical complexity. J Pediatr Rehabil Med. 2015;8(2):83-95. doi:10.3233/PRM-150322 |
| 1. Domingo B, Terroso N, Eckert M. Personalized Use of an Adjustable Movement-Controlled Video Game in Obstetric Brachial Plexus Palsy during Physiotherapy Sessions at School: A Case Report. Healthcare (Basel). 2023;11(14)doi:10.3390/healthcare11142008 |
| 1. Dos Santos Alves VL, Stirbulov R, Avanzi O. Long-term impact of pre-operative physical rehabilitation protocol on the 6-min walk test of patients with adolescent idiopathic scoliosis: A randomized clinical trial. Rev Port Pneumol (2006). 2015;21(3):138-43. doi:10.1016/j.rppnen.2014.08.006 |
| 1. Duarte NdAC, Grecco LAC, Galli M, Fregni F, Oliveira CS. Effect of transcranial direct-current stimulation combined with treadmill training on balance and functional performance in children with cerebral palsy: a double-blind randomized controlled trial. PloS one. 2014;9(8):e105777. doi:10.1371/journal.pone.0105777 |
| 1. Dufvenberg M, Diarbakerli E, Charalampidis A, et al. Six-Month Results on Treatment Adherence, Physical Activity, Spinal Appearance, Spinal Deformity, and Quality of Life in an Ongoing Randomised Trial on Conservative Treatment for Adolescent Idiopathic Scoliosis (CONTRAIS). J Clin Med. 2021;10(21)doi:10.3390/jcm10214967 |
| 1. Dulfer K, Duppen N, Kuipers IM, et al. Aerobic exercise influences quality of life of children and youngsters with congenital heart disease: a randomized controlled trial. J Adolesc Health. 2014;55(1):65-72. doi:10.1016/j.jadohealth.2013.12.010 |
| 1. Dunlap PM, Mucha A, Smithnosky D, et al. The Gaze Stabilization Test Following Concussion. J Am Acad Audiol. 2018;doi:10.3766/jaaa.18015 |
| 1. Ehrenborg C, Gustafsson S, Archenholtz B. Long-term effect in ADL after an interdisciplinary rehabilitation programme for WAD patients: a mixed-method study for deeper understanding of participants' programme experiences. Disabil Rehabil. 2014;36(12):1006-13. doi:10.3109/09638288.2013.825651 |
| 1. Elnaggar RK, Mahmoud WS, Abdelbasset WK, Alqahtani BA, Alrawaili SM, Elfakharany MS. Low-energy laser therapy application on knee joints as an auxiliary treatment in patients with polyarticular juvenile idiopathic arthritis: a dual-arm randomized clinical trial. Lasers Med Sci. 2022;37(3):1737-1746. doi:10.1007/s10103-021-03427-6 |
| 1. Ferrari A, Maoret AR, Muzzini S, et al. A randomized trial of upper limb botulimun toxin versus placebo injection, combined with physiotherapy, in children with hemiplegia. Res Dev Disabil. 2014;35(10):2505-13. doi:10.1016/j.ridd.2014.06.001 |
| 1. Figueiredo PRP, Mancini MC, Feitosa AM, et al. Hand-arm bimanual intensive therapy and daily functioning of children with bilateral cerebral palsy: a randomized controlled trial. Dev Med Child Neurol. 2020;62(11):1274-1282. doi:10.1111/dmcn.14630 |
| 1. Filbay S, Kvist J. Fear of Reinjury Following Surgical and Nonsurgical Management of Anterior Cruciate Ligament Injury: An Exploratory Analysis of the NACOX Multicenter Longitudinal Cohort Study. Phys Ther. 2022;102(2)doi:10.1093/ptj/pzab273 |
| 1. Filipa A, Barton K. Physical Therapy Rehabilitation of an Adolescent Preprofessional Dancer Following Os Trigonum Excision: A Case Report. J Orthop Sports Phys Ther. 2018;48(3):194-203. doi:10.2519/jospt.2018.7508 |
| 1. Fisher-Pipher SPD, Kenyon LKPDPP, Westman MPD. Improving balance, mobility, and dual-task performance in an adolescent with cerebral palsy: A case report. Physiother Theory Pract. 2017;33(7):586-595. doi:10.1080/09593985.2017.1323359 |
| 1. Fourman MS, Hassan SG, Roach JW, Grudziak JS. Anatomic all-epiphyseal ACL reconstruction with "inside-out" femoral tunnel placement in immature patients yields high return to sport rates and functional outcome scores a minimum of 24 months after reconstruction. Knee Surg Sports Traumatol Arthrosc. 2021;29(12):4251-4260. doi:10.1007/s00167-021-06542-7 |
| 1. Franki I, Desloovere K, De Cat J, et al. An evaluator-blinded randomized controlled trial evaluating therapy effects and prognostic factors for a general and an individually defined physical therapy program in ambulant children with bilateral spastic cerebral palsy. Eur J Phys Rehabil Med. 2015;51(6):677-91. |
| 1. Franklin AD, Cierny GB, Luckett TR. Interventional and multimodal pain rehabilitation in a child with meralgia paresthetica. J Clin Anesth. 2016;33:456-9. doi:10.1016/j.jclinane.2016.04.015 |
| 1. Friel KM, Kuo H-C, Fuller J, et al. Skilled Bimanual Training Drives Motor Cortex Plasticity in Children With Unilateral Cerebral Palsy. Neurorehabil Neural Repair. 2016;30(9):834-44. doi:10.1177/1545968315625838 |
| 1. Gao S, Treble-Barna A, Fabio A, et al. Effects of inpatient rehabilitation after acute care on functional and quality-of-life outcomes in children with severe traumatic brain injury. Brain Inj. 2022;36(10):1280-1287. doi:10.1080/02699052.2022.2120211 |
| 1. Gauntlett-Gilbert J, Bhat C, Clinch J. Body mass in adolescents with chronic pain: observational study. Arch Dis Child. 2020;105(5):476-480. doi:10.1136/archdischild-2019-317843 |
| 1. Gauvin-Lepage J, Friedman D, Grilli L, Sufrategui M, De Matteo C, Iverson GL, Gagnon I. Effectiveness of an Exercise-Based Active Rehabilitation Intervention for Youth Who Are Slow to Recover After Concussion. Clin J Sport Med. 2020;30(5):423-432. doi:10.1097/JSM.0000000000000634 |
| 1. Gibson N, Chappell A, Blackmore AM, Morris S, Williams G, Bear N, Allison G. The effect of a running intervention on running ability and participation in children with cerebral palsy: a randomized controlled trial. Disabil Rehabil. 2018;40(25):3041-3049. doi:10.1080/09638288.2017.1367426 |
| 1. Gilardi F, De Falco F, Casasanta D, et al. Robotic Technology in Pediatric Neurorehabilitation. A Pilot Study of Human Factors in an Italian Pediatric Hospital. Int J Environ Res Public Health. 2020;17(10)doi:10.3390/ijerph17103503 |
| 1. Gomes ELFD, Carvalho CRF, Peixoto-Souza FS, et al. Active Video Game Exercise Training Improves the Clinical Control of Asthma in Children: Randomized Controlled Trial. PloS one. 2015;10(8):e0135433. doi:10.1371/journal.pone.0135433 |
| 1. Goyal C, Vardhan V, Naqvi W. Non-Immersive Virtual Reality as an Intervention for Improving Hand Function and Functional Independence in Children With Unilateral Cerebral Palsy: A Feasibility Study. Cureus. 2022;14(6):e26085. doi:10.7759/cureus.26085 |
| 1. Goyal C, Vardhan V, Naqvi WM. Haptic Feedback-Based Virtual Reality Intervention for a Child With Infantile Hemiplegia: A Case Report. Cureus. 2022;14(3):e23489. doi:10.7759/cureus.23489 |
| 1. Grecco LAC, Oliveira CS, Duarte NdAC, Lima VLCC, Zanon N, Fregni F. Cerebellar transcranial direct current stimulation in children with ataxic cerebral palsy: A sham-controlled, crossover, pilot study. Dev Neurorehabil. 2017;20(3):142-148. doi:10.3109/17518423.2016.1139639 |
| 1. Grecco LAC, Zanon N, Sampaio LMM, Oliveira CS. A comparison of treadmill training and overground walking in ambulant children with cerebral palsy: randomized controlled clinical trial. Clin Rehabil. 2013;27(8):686-96. doi:10.1177/0269215513476721 |
| 1. Griffin A, Wilson L, Feinstein AB, et al. Virtual Reality in Pain Rehabilitation for Youth With Chronic Pain: Pilot Feasibility Study. JMIR Rehabil Assist Technol. 2020;7(2):e22620. doi:10.2196/22620 |
| 1. Griffin DR, Dickenson EJ, Achana F, et al. Arthroscopic hip surgery compared with personalised hip therapy in people over 16 years old with femoroacetabular impingement syndrome: UK FASHIoN RCT. Health Technol Assess. 2022;26(16):1-236. doi:10.3310/FXII0508 |
| 1. Grinde K, Myhre J, Finch MD. Repeated episodes of pediatric constraint induced movement therapy with a gross motor training component: A prospective cohort study. J Pediatr Rehabil Med. 2020;13(2):149-159. doi:10.3233/PRM-180543 |
| 1. Grodon C, Bassett P, Shannon H. The 'heROIC' trial: Does the use of a robotic rehabilitation trainer change quality of life, range of movement and function in children with cerebral palsy? Child Care Health Dev. 2023;49(5):914-924. doi:10.1111/cch.13101 |
| 1. Grosse L, Spah MA, Borner C, et al. Addressing gross motor function by functional repetitive neuromuscular magnetic stimulation targeting to the gluteal muscles in children with bilateral spastic cerebral palsy: benefits of functional repetitive neuromuscular magnetic stimulation targeting the gluteal muscles. Front Neurol. 2023;14:1161532. doi:10.3389/fneur.2023.1161532 |
| 1. Hazany S, Bagrodia N, Chu R, Jr., Shaw S. Results of a 2-week novel robotic rehabilitation program in 18 children with prior hemispherectomy. J Clin Neurosci. 2023;108:6-12. doi:10.1016/j.jocn.2022.12.011 |
| 1. Hepworth C, Sinha I, Saint GL, Hawcutt DB. Assessing the impact of breathing retraining on asthma symptoms and dysfunctional breathing in children. Pediatr Pulmonol. 2019;54(6):706-712. doi:10.1002/ppul.24300 |
| 1. Holm S, Ljungman G, Asenlof P, Linton SJ, Soderlund A. Treating youth in pain: Comparing tailored behavioural medicine treatment provided by physical therapists in primary care with physical exercises. Eur J Pain. 2016;20(4):626-38. doi:10.1002/ejp.780 |
| 1. Hsieh R-L, Lee W-C, Lin J-H. The Impact of Short-Term Video Games on Performance among Children with Developmental Delays: A Randomized Controlled Trial. PloS one. 2016;11(3):e0149714. doi:10.1371/journal.pone.0149714 |
| 1. Jackman T, Devine N, Seiger C. Home health physical therapy intervention for an adolescent after intramedullary skeletal kinetic distractor limb-lengthening surgery: a case report. J Allied Health. 2013;42(3):163-8. |
| 1. Johanson ME, Jaramillo JP, Dairaghi CA, Murray WM, Hentz VR. Multicenter Survey of the Effects of Rehabilitation Practices on Pinch Force Strength After Tendon Transfer to Restore Pinch in Tetraplegia. Arch Phys Med Rehabil. 2016;97(6):S105-16. doi:1016/j.apmr.2016.01.036 |
| 1. Jorgensen SL, Mechlenburg I. Effects of Low-Load Blood-Flow Restricted Resistance Training on Functional Capacity and Patient-Reported Outcome in a Young Male Suffering From Reactive Arthritis. Front Sports Act Living. 2021;3:798902. doi:10.3389/fspor.2021.798902 |
| 1. Joshi S, Parmar S, Kalavant A, Shah L, Parmar D. Effectiveness of structured physiotherapy in constipation in children with neurodevelopmental disorders-a randomized trial. Physiother Theory Pract. 2022:1-9. doi:10.1080/09593985.2022.2100299 |
| 1. Kara OK, Yardimci BN, Sahin S, Orhan C, Livanelioglu A, Soylu AR. Combined Effects of Mirror Therapy and Exercises on the Upper Extremities in Children with Unilateral Cerebral Palsy: A Randomized Controlled Trial. Dev Neurorehabil. 2020;23(4):253-264. doi:10.1080/17518423.2019.1662853 |
| 1. Karibe H, Goddard G, Shimazu K, Kato Y, Warita-Naoi S, Kawakami T. Comparison of self-reported pain intensity, sleeping difficulty, and treatment outcomes of patients with myofascial temporomandibular disorders by age group: a prospective outcome study. BMC Musculoskelet Disord. 2014;15:423. doi:10.1186/1471-2474-15-423 |
| 1. Karol LA, Jeans KA, Kaipus KA. The Relationship Between Gait, Gross Motor Function, and Parental Perceived Outcome in Children With Clubfeet. J Pediatr Orthop. 2016;36(2):145-51. doi:10.1097/BPO.0000000000000410 |
| 1. Kempert H, Benore E, Heines R. Physical and occupational therapy outcomes: Adolescents' change in functional abilities using objective measures and self-report. Scand J Pain. 2017;14:60-66. doi:10.1016/j.sjpain.2016.10.004 |
| 1. Kepenek-Varol B, Hosbay Z. Is short-term hand therapy effective in a child with congenital radioulnar synostosis? A case report. J Hand Ther. 2020;33(3):435-442. doi:10.1016/j.jht.2019.03.009 |
| 1. Kim M, Park C, Jeon H, Choi WJ, You SJH. Comparative effects of community-based family-child-centered care and conventional pediatric rehabilitation for cerebral palsy. NeuroRehabilitation. 2021;49(4):533-546. doi:10.3233/NRE-210219 |
| 1. Klingels K, Feys H, Molenaers G, et al. Randomized trial of modified constraint-induced movement therapy with and without an intensive therapy program in children with unilateral cerebral palsy. Neurorehabil Neural Repair. 2013;27(9):799-807. doi:10.1177/1545968313496322 |
| 1. Ko EJ, Sung IY, Moon HJ, Yuk JS, Kim H-S, Lee NH. Effect of Group-Task-Oriented Training on Gross and Fine Motor Function, and Activities of Daily Living in Children with Spastic Cerebral Palsy. Phys Occup Ther Pediatr. 2020;40(1):18-30. doi:10.1080/01942638.2019.1642287 |
| 1. Kotte EMW, de Groot JF, Winkler AMF, Huijgen BCH, Takken T. Effects of the Fitkids exercise therapy program on health-related fitness, walking capacity, and health-related quality of life. Phys Ther. 2014;94(9):1306-18. doi:10.2522/ptj.20130315 |
| 1. Kraft KA, Weisberg J, Finch MD, Nickel A, Griffin KH, Barnes TL. Hippotherapy in Rehabilitation Care for Children With Neurological Impairments and Developmental Delays: A Case Series. Pediatr Phys Ther. 2019;31(1):E14-E21. doi:10.1097/PEP.0000000000000567 |
| 1. Krasovsky T, Barak S, Silberg T, Yissar T, Brezner A, Landa J. Factors Associated With Gains in Performance During Rehabilitation After Pediatric Brain Injury: Growth Curve Analysis. Am J Phys Med Rehabil. 2020;99(4):310-317. doi:10.1097/PHM.0000000000001329 |
| 1. Kroll KH, Kovach JR, Ginde S, Jacobsen RM, Danduran M, Foster A, Brosig CL. Impact of a paediatric cardiac rehabilitation programme upon patient quality of life. Cardiol Young. 2021;31(5):804-811. doi:10.1017/S1047951120004710 |
| 1. Kushnir A, Kachmar O. Intensive Neurophysiological Rehabilitation System for children with cerebral palsy: a quasi-randomized controlled trial. BMC Neurol. 2023;23(1):157. doi:10.1186/s12883-023-03216-4 |
| 1. Lantz JM, Joshi S, O'Hearn M. The Effects of Orthopedic Manual Physical Therapy in the Management of Juvenile Idiopathic Arthritis: A Case Report. Pediatr Phys Ther. 2016;28(4):490-7. doi:10.1097/PEP.0000000000000319 |
| 1. Lauglo R, Vik T, Lamvik T, Stensvold D, Finbraten A-K, Moholdt T. High-intensity interval training to improve fitness in children with cerebral palsy. BMJ Open Sport Exerc Med. 2016;2(1):e000111. doi:10.1136/bmjsem-2016-000111 |
| 1. Ledger SJ, Owen E, Prasad SA, Goldman A, Willams J, Aurora P. A pilot outreach physiotherapy and dietetic quality improvement initiative reduces IV antibiotic requirements in children with moderate-severe cystic fibrosis. J Cyst Fibros. 2013;12(6):766-72. doi:10.1016/j.jcf.2013.01.003 |
| 1. Levy TJ, Tyner CE, Amaral S, Lefkowitz DS, Kessler SK, Levin LS. 5-Year Activity and Participation Outcomes of the First Successful Pediatric Bilateral Hand Transplantation: A Case Report. Phys Occup Ther Pediatr. 2022;42(6):663-679. doi:10.1080/01942638.2022.2057210 |
| 1. Li W, Liu T, Yao M, Yu R, Shu M, Zhang M, Huang J. Effect of interesting respiratory rehabilitation training for the treatment of refractory Mycoplasma pneumoniae pneumonia in children. BMC Infect Dis. 2023;23(1):561. doi:10.1186/s12879-023-08513-4 |
| 1. Logan DE, Sieberg CB, Conroy C, Smith K, Odell S, Sethna N. Changes in sleep habits in adolescents during intensive interdisciplinary pediatric pain rehabilitation. J Youth Adolesc. 2015;44(2):543-55. doi:10.1007/s10964-014-0155-2 |
| 1. Louw A, Puentedura EJ, Reese D, Parker P, Miller T, Mintken PE. Immediate Effects of Mirror Therapy in Patients With Shoulder Pain and Decreased Range of Motion. Arch Phys Med Rehabil. 2017;98(10):1941-1947. doi:10.1016/j.apmr.2017.03.031 |
| 1. Maharaj SS, Lallie R. Does a physiotherapy programme of gross motor training influence motor function and activities of daily living in children presenting with developmental coordination disorder? S Afr J Physiother. 2016;72(1):304. doi:10.4102/sajp.v72i1.304 |
| 1. Meghe S, Chitale N, Phansopkar P, Joshi A. Effectiveness of Early Physical Therapy Rehabilitation in Patient With Juvenile Rheumatoid Arthritis. Cureus. 2022;14(10):e30213. doi:10.7759/cureus.30213 |
| 1. Mendonca TM, Terreri MT, Silva CH, Neto MB, Pinto RM, Natour J, Len CA. Effects of Pilates exercises on health-related quality of life in individuals with juvenile idiopathic arthritis. Arch Phys Med Rehabil. 2013;94(11):2093-102. doi:10.1016/j.apmr.2013.05.026 |
| 1. Menz SM, Hatten K, Grant-Beuttler M. Strength training for a child with suspected developmental coordination disorder. Pediatr Phys Ther. 2013;25(2):214-23. doi:10.1097/PEP.0b013e31828a2042 |
| 1. Mirek E, Logan D, Boullard K, Hall AM, Staffa SJ, Sethna N. Physical Therapy Outcome Measures for Assessment of Lower Extremity Chronic Pain-Related Function in Pediatrics. Pediatr Phys Ther. 2019;31(2):200-207. doi:10.1097/PEP.0000000000000587 |
| 1. Mishra K, Siddharth V, Elhence A, Jalan D, Khera D, Yasir M. Effectiveness of individualized, goal directed institutional based rehabilitation programme in children with developmental delay disorders, in a region with limited rehabilitation accessibility. J Pediatr Rehabil Med. 2019;12(1):49-56. doi:10.3233/PRM-170521 |
| 1. Moawd SA, Azab AR, Ibrahim ZM, Verma A, Abdelbasset WK. Impacts of Respiratory Muscle Training on Respiratory Functions, Maximal Exercise Capacity, Functional Performance, and Quality of Life in School-Aged Children with Postoperative Congenital Diaphragmatic Hernia. Dis Markers. 2020;2020:8829373. doi:10.1155/2020/8829373 |
| 1. Montpetit K, Palomo T, Glorieux FH, Fassier F, Rauch F. Multidisciplinary Treatment of Severe Osteogenesis Imperfecta: Functional Outcomes at Skeletal Maturity. Arch Phys Med Rehabil. 2015;96(10):1834-9. doi:10.1016/j.apmr.2015.06.006 |
| 1. Muller C, Krauth KA, Gers J, Rosenbaum D. Physical activity and health-related quality of life in pediatric cancer patients following a 4-week inpatient rehabilitation program. Support Care Cancer. 2016;24(9):3793-802. doi:10.1007/s00520-016-3198-y |
| 1. Nangliya RM, Jain DS, Saklecha AV, Patil DS. Effect of physiotherapy rehabilitation on osteogenesis imperfecta with a midshaft tibial fracture in the 11-year-old patient: a case report. Pan Afr Med J. 2022;43:201. doi:10.11604/pamj.2022.43.201.34702 |
| 1. Nelson S, Smith AM, Jervis K, Silvia MN, Randall E. Will This Treatment Help My Child?: How Parent/Caregiver Treatment Expectations Relate to Intensive Pain Rehabilitation Outcomes for Youth With Chronic Pain. Clin J Pain. 2022;38(11):651-658. doi:10.1097/AJP.0000000000001068 |
| 1. Noordstar JJ, van der Net J, Voerman L, Helders PJM, Jongmans MJ. The effect of an integrated perceived competence and motor intervention in children with developmental coordination disorder. Res Dev Disabil. 2017;60:162-175. doi:10.1016/j.ridd.2016.12.002 |
| 1. O'Keeffe S, Ni Cheilleachair N, O'Connor S. Fear Avoidance Following Musculoskeletal Injury in Male Adolescent Gaelic Footballers. J Sport Rehabil. 2020;29(4):413-419. doi:10.1123/jsr.2018-0258 |
| 1. Okur EO, Arik MI, Okur I, Gokpinar HH, Gunel MK. Dual-task training effect on gait parameters in children with spastic diplegic cerebral palsy: Preliminary results of a self-controlled study. Gait Psture. 2022;94:45-50. doi:10.1016/j.gaitpost.2022.02.020 |
| 1. Ortega Solis J, Reynard P, Spruyt K, Becaud C, Ionescu E, Thai-Van H. Developing a serious game for gaze stability rehabilitation in children with vestibular hypofunction. J Neuroeng Rehabil. 2023;20(1):128. doi:10.1186/s12984-023-01249-x |
| 1. Osborn H, Reek S, Anderson B. Interdisciplinary Occupational and Physical Therapy Approach to Treating Constipation and Fecal Incontinence in Children. Phys Occup Ther Pediatr. 2023;43(2):243-256. doi:10.1080/01942638.2022.2131499 |
| 1. Ovans JA, Hooke MC, Bendel AE, Tanner LR. Physical Therapist Coaching to Improve Physical Activity in Children With Brain Tumors: A Pilot Study. Pediatr Phys Ther. 2018;30(4):310-317. doi:10.1097/PEP.0000000000000531 |
| 1. Pacey V, Tofts L, Adams RD, Munns CF, Nicholson LL. Exercise in children with joint hypermobility syndrome and knee pain: a randomised controlled trial comparing exercise into hypermobile versus neutral knee extension. Pediatr Rheumatol Online J. 2013;11(1):30. doi:10.1186/1546-0096-11-30 |
| 1. Parry I, Painting L, Bagley A, et al. A Pilot Prospective Randomized Control Trial Comparing Exercises Using Videogame Therapy to Standard Physical Therapy: 6 Months Follow-Up. J Burn Care Res. 2015;36(5):534-44. doi:10.1097/BCR.0000000000000165 |
| 1. Perets I, Hartigan DE, Chaharbakhshi EO, Ashberg L, Mu B, Domb BG. Clinical Outcomes and Return to Sport in Competitive Athletes Undergoing Arthroscopic Iliopsoas Fractional Lengthening Compared With a Matched Control Group Without Iliopsoas Fractional Lengthening. Arthroscopy. 2018;34(2):456-463. doi:10.1016/j.arthro.2017.08.292 |
| 1. Ramey SL, DeLuca SC, Stevenson RD, Conaway M, Darragh AR, Lo W. Constraint-Induced Movement Therapy for Cerebral Palsy: A Randomized Trial. Pediatrics. 2021;148(5)doi:10.1542/peds.2020-033878 |
| 1. Rasmussen HM, Pedersen NW, Overgaard S, et al. Gait analysis for individually tailored interdisciplinary interventions in children with cerebral palsy: a randomized controlled trial. Dev Med Child Neurol. 2019;61(10):1189-1195. doi:10.1111/dmcn.14178 |
| 1. Rastgar Koutenaei F, Noorizadeh Dehkordi S, Amini M, ShahAli S. Effect of Swiss Ball Stabilization Training on Trunk Control, Abdominal Muscle Thickness, Balance, and Motor Skills of Children With Spastic Cerebral Palsy: A Randomized, Superiority Trial. Arch Phys Med Rehabil. 2023;doi:10.1016/j.apmr.2023.05.011 |
| 1. Rathleff MS, Graven-Nielsen T, Holmich P, Winiarski L, Krommes K, Holden S, Thorborg K. Activity Modification and Load Management of Adolescents With Patellofemoral Pain: A Prospective Intervention Study Including 151 Adolescents. Am J Sports Med. 2019;47(7):1629-1637. doi:10.1177/0363546519843915 |
| 1. Rathleff MS, Rathleff CR, Holden S, Thorborg K, Olesen JL. Exercise therapy, patient education, and patellar taping in the treatment of adolescents with patellofemoral pain: a prospective pilot study with 6 months follow-up. Pilot Feasibility Stud. 2018;4:73. doi:10.1186/s40814-017-0227-7 |
| 1. Rathleff MS, Roos EM, Olesen JL, Rasmussen S. Exercise during school hours when added to patient education improves outcome for 2 years in adolescent patellofemoral pain: a cluster randomised trial. Br J Sports Med. 2015;49(6):406-12. doi:10.1136/bjsports-2014-093929 |
| 1. Reedman SE, Boyd RN, Trost SG, Elliott C, Sakzewski L. Efficacy of Participation-Focused Therapy on Performance of Physical Activity Participation Goals and Habitual Physical Activity in Children With Cerebral Palsy: A Randomized Controlled Trial. Arch Phys Med Rehabil. 2019;100(4):676-686. doi:10.1016/j.apmr.2018.11.012 |
| 1. Reubens R, Silkwood-Sherer DJ. Intervention for an Adolescent With Cerebral Palsy During Period of Accelerated Growth. Pediatr Phys Ther. 2016;28(1):117-25. doi:10.1097/PEP.0000000000000223 |
| 1. Riedl D, Licht T, Nickels A, et al. Large Improvements in Health-Related Quality of Life and Physical Fitness during Multidisciplinary Inpatient Rehabilitation for Pediatric Cancer Survivors. Cancers. 2022;14(19)doi:10.3390/cancers14194855 |
| 1. Ryan JL, Zhou C, Levac DE, Fehlings DL, Beal DS, Hung R, Wright FV. Gross motor change after inpatient rehabilitation for children with acquired brain injury: A 10-year retrospective review. Dev Med Child Neurol. 2023;65(7):953-960. doi:10.1111/dmcn.15471 |
| 1. Schasfoort F, Pangalila R, Sneekes EM, et al. Intramuscular botulinum toxin prior to comprehensive rehabilitation has no added value for improving motor impairments, gait kinematics and goal attainment in walking children with spastic cerebral palsy. J Rehabil Med. 2018;50(8):732-742. doi:10.2340/16501977-2369 |
| 1. Scheffers LE, Somers OC, Dulfer K, et al. Physical training and high-protein diet improved muscle strength, parent-reported fatigue, and physical quality of life in children with Pompe disease. J Inherit Metab Dis. 2023;46(4):605-617. doi:10.1002/jimd.12607 |
| 1. Senn-Malashonak A, Wallek S, Schmidt K, Rosenhagen A, Vogt L, Bader P, Banzer W. Psychophysical effects of an exercise therapy during pediatric stem cell transplantation: a randomized controlled trial. Bone Marrow Transplant. 2019;54(11):1827-1835. doi:10.1038/s41409-019-0535-z |
| 1. Sherry DD, Brake L, Tress JL, Sherker J, Fash K, Ferry K, Weiss PF. The Treatment of Juvenile Fibromyalgia with an Intensive Physical and Psychosocial Program. J Pediatr. 2015;167(3):731-7. doi:10.1016/j.jpeds.2015.06.036 |
| 1. Silva T, Tobelem DdC, Malavazzi TCDS, et al. Effect of Photobiomodulation Combined with Physiotherapy on Functional Performance in Children with Myelomeningo-Cele-Randomized, Blind, Clinical Trial. J Clin Med. 2023;12(8)doi:10.3390/jcm12082920 |
| 1. Simons LE, Sieberg CB, Pielech M, Conroy C, Logan DE. What does it take? Comparing intensive rehabilitation to outpatient treatment for children with significant pain-related disability. J Pediatr Psychol. 2013;38(2):213-23. doi:10.1093/jpepsy/jss109 |
| 1. Smith C, Farhat R, Fern-Buneo A, et al. Effects of an exercise program during pediatric stem cell transplantation: A randomized controlled trial. Pediatr Blood Cancer. 2022;69(5):e29618. doi:10.1002/pbc.29618 |
| 1. Smith HJ. Physical therapy for a child with sudden-onset choreoathetosis: a case report. Pediatr Phys Ther. 2014;26(1):85-93. doi:10.1097/PEP.0000000000000010 |
| 1. Soee A-BL, Skov L, Skovgaard LT, Thomsen LL. Headache in children: effectiveness of multidisciplinary treatment in a tertiary paediatric headache clinic. Cephalalgia. 2013;33(15):1218-28. doi:10.1177/0333102413490349 |
| 1. Speth L, Janssen-Potten Y, Rameckers E, et al. Effects of botulinum toxin A and/or bimanual task-oriented therapy on upper extremity activities in unilateral Cerebral Palsy: a clinical trial. BMC Neurol. 2015;15:143. doi:10.1186/s12883-015-0404-3 |
| 1. Straume-Naesheim TM, Randsborg P-H, Mikaelsen JR, Aroen A. Medial patellofemoral ligament reconstruction is superior to active rehabilitation in protecting against further patella dislocations. Knee Surg Sports Traumatol Arthrosc. 2022;30(10):3428-3437. doi:10.1007/s00167-022-06934-3 |
| 1. Stromback M, Wiklund M, Salander Renberg E, Malmgren-Olsson E-B. Gender-sensitive and youth-friendly physiotherapy: Steps toward a stress management intervention for girls and young women. Physiother Theory Pract. 2016;32(1):20-33. doi:10.3109/09593985.2015.1075639 |
| 1. Summers J, Coker B, Eddy S, et al. Selective dorsal rhizotomy in ambulant children with cerebral palsy: an observational cohort study. Lancet Child Adolesc Health. 2019;3(7):455-462. doi:10.1016/S2352-4642(19)30119-1 |
| 1. Tarakci E, Arman N, Tarakci D, Kasapcopur O. Leap Motion Controller-based training for upper extremity rehabilitation in children and adolescents with physical disabilities: A randomized controlled trial. J Hand Ther. 2020;33(2):220-228.e1. doi:10.1016/j.jht.2019.03.012 |
| 1. Tezcan S, Cankaya T. The effect of modified constraint-induced movement therapy in children with hemiparetic cerebral palsy. Consecutive or intermittent days? Disabil Rehabil. 2022;44(24):7500-7507. doi:10.1080/09638288.2021.2002441 |
| 1. Thomas RE, Johnston LM, Sakzewski L, Kentish MJ, Boyd RN. Evaluation of group versus individual physiotherapy following lower limb intra-muscular Botulinum Toxin-Type A injections for ambulant children with cerebral palsy: A single-blind randomized comparison trial. Res Dev Disabil. 2016;53:267-78. doi:10.1016/j.ridd.2016.02.014 |
| 1. Thorsteinsdottir H, Diseth TH, Lie A, Tangeraas T, Matthews I, Asberg A, Bjerre A. Small effort, high impact: Focus on physical activity improves oxygen uptake (VO2peak ), quality of life, and mental health after pediatric renal transplantation. Pediatr Transplant. 2018:e13242. doi:10.1111/petr.13242 |
| 1. Toovey RAM, Harvey AR, McGinley JL, Lee KJ, Shih STF, Spittle AJ. Task-specific training for bicycle-riding goals in ambulant children with cerebral palsy: a randomized controlled trial. Dev Med Child Neurol. 2022;64(2):243-252. doi:10.1111/dmcn.15029 |
| 1. Tsao E, Flanigan M, Johnson L, Toy M, Giannini E, Bjornson K. Functional outcomes of pediatric patients in Short-term Pediatric Rehabilitation Intensive Therapy (SPRINT) while receiving acute oncologic and hematologic care. PM & R. 2022;14(3):357-365. doi:10.1002/pmrj.12595 |
| 1. Tucker P. Effectiveness of Robotic-Assisted Gait Training and Aquatic Physical Therapy in a Child With Long-Chain 3-Hydroxyacyl-CoA Dehydrogenase Deficiency: A Case Report. Pediatr Phys Ther. 2022;34(4):563-569. doi:10.1097/PEP.0000000000000951 |
| 1. Ullenhag A, Granlund M, Almqvist L, Krumlinde-Sundholm L. A Strength-Based Intervention to Increase Participation in Leisure Activities in Children with Neuropsychiatric Disabilities: A Pilot Study. Occup Ther Int. 2020;2020:1358707. doi:10.1155/2020/1358707 |
| 1. Unes S, Tuncdemir M, Eroglu-Ertugrul NG, Kerem Gunel M. Effectiveness of Physical Therapy on Ataxia-Telangiectasia: A Case Report. Pediatr Phys Ther. 2021;33(3):E103-E107. doi:10.1097/PEP.0000000000000813 |
| 1. Van der Looven R, De Vos E, Vandekerckhove K, Coomans I, Laureys G, Dhooge C. Efficacy of interdisciplinary rehabilitation in child cancer survivors: Impact on physical fitness, fatigue and body composition after 1-year follow-up. Eur J Cancer Care (Engl). 2022;31(6):e13761. doi:10.1111/ecc.13761 |
| 1. van Dijk-Lokkart EM, Braam KI, van Dulmen-den Broeder E, et al. Effects of a combined physical and psychosocial intervention program for childhood cancer patients on quality of life and psychosocial functioning: results of the QLIM randomized clinical trial. Psychooncology. 2016;25(7):815-22. doi:10.1002/pon.4016 |
| 1. van Engelenburg-van Lonkhuyzen ML, Bols EMJ, Benninga MA, Verwijs WA, de Bie RA. Effectiveness of Pelvic Physiotherapy in Children With Functional Constipation Compared With Standard Medical Care. Gastroenterology. 2017;152(1):82-91. doi:10.1053/j.gastro.2016.09.015 |
| 1. Van Meulenbroek T, Conijn AEA, Huijnen IPJ, Engelbert RHH, Verbunt JA. Multidisciplinary Treatment for Hypermobile Adolescents with Chronic Musculoskeletal Pain. J Rehabil Med Clin Commun. 2020;3:1000033. doi:10.2340/20030711-1000033 |
| 1. Van Wely L, Balemans AC, Becher JG, Dallmeijer AJ. Physical activity stimulation program for children with cerebral palsy did not improve physical activity: a randomised trial. J Physiother. 2014;60(1):40-9. doi:10.1016/j.jphys.2013.12.007 |
| 1. Vuorimaa H, Leppanen L, Kautiainen H, Mikkelsson M, Hietanen M, Vilen H, Pohjankoski H. Risk severity moderated effectiveness of pain treatment in adolescents. Scand J Pain. 2019;19(2):287-298. doi:10.1515/sjpain-2018-0312 |
| 1. Wiegerinck JI, Zwiers R, Sierevelt IN, van Weert HCPM, van Dijk CN, Struijs PAA. Treatment of Calcaneal Apophysitis: Wait and See Versus Orthotic Device Versus Physical Therapy: A Pragmatic Therapeutic Randomized Clinical Trial. J Pediatr Orthop. 2016;36(2):152-7. doi:10.1097/BPO.0000000000000417 |
| 1. Winkels DGM, Kottink AIR, Temmink RAJ, Nijlant JMM, Buurke JH. Wii TM-habilitation of upper extremity function in children with cerebral palsy. An explorative study. Dev Neurorehabil. 2013;16(1):44-51. doi:10.3109/17518423.2012.713401 |
| 1. Yigit S, Albayrak HM, Perk Yucel P, Usgu S, Yakut Y. The Outcomes of an Individualized Physical Therapy Program in CALFAN Syndrome: A Case Report. Pediatr Phys Ther. 2022;34(3):432-437. doi:10.1097/PEP.0000000000000903 |
| 1. Zogby AM, Bomar JD, Johnson KP, Upasani VV, Pennock AT. Nonoperative Management of Femoroacetabular Impingement in Adolescents: Clinical Outcomes at a Mean of 5 Years: A Prospective Study. Am J Sports Med. 2021;49(11):2960-2967. doi:10.1177/03635465211030512 |
