# Supplementary material for: Patient reported outcomes in pediatric physical therapy: a scoping review and evidence map
Source: J Patient Rep Outcomes. 2025 Oct 24;9:125. doi: 10.1186/s41687-025-00947-5 (PMC12552199; doi:10.1186/s41687-025-00947-5)
Supplement: Supplementary file 4 — Supplementary Material 4 [file 41687_2025_947_MOESM4_ESM.docx]

**Additional file 4: PROMs used in the included studies**

| **Patient Reported Outcome Measure (PROM)** | **Abbreviation of PROM** | **Number of included records in which PROM was used** |
| --- | --- | --- |
| Canadian Occupational Performance Measure | COPM | 28 |
| Pediatric Quality of Life Inventory | PedsQL | 27 |
| Pediatric Evaluation of Disability Inventory | PEDI | 24 |
| pain visual analogue scale | pain VAS | 19 |
| Numeric Pain Rating Scale | NPRS | 13 |
| Gait Outcomes Assessment List | GAS | 12 |
| ABILHAND-Kids | ABILHAND-Kids | 11 |
| Functional Disability Inventory | FDI | 8 |
| Childhood Health Assessment Questionnaire | CHAQ | 7 |
| Pain Catastrophization Scale | PCS | 7 |
| Knee Self-Efficacy Scale | KOOS | 6 |
| Patient Reported Outcome Measure created for the specific research in which is was used | self-created | 6 |
| Childen's Depression Inventory | CDI | 5 |
| EuroQol 5D | EQ-5D | 5 |
| Pediatric Evaluation of Disability Inventory Computerized Adaptive Test | PEDI-CAT | 5 |
| Pediatric Outcomes Data Collection Instrument | PODCI | 5 |
| Children’s Assessment of Participation and Enjoyment | CAPE | 4 |
| Developmental Coordination Disorder Questionnaire | DCD-Q | 4 |
| Borg’s rating of perceived exertion | Borg | 3 |
| Bristol Stool Form Scale | BSFS | 3 |
| Center for Epidemiological Studies Depression Scale for Children | CES-DC | 3 |
| Children’s Hand-use Experience Questionnaire | CHEQ | 3 |
| Fatigue Severity Scale | FPS | 3 |
| Knee injury and Osteoarthritis Outcome Score | KINDL | 3 |
| Lower Extremity Functional Scale | LEFS | 3 |
| Patient Specific Functional Scale | PSFS | 3 |
| Self-Perception Profile | SPP | 3 |
| 36-Item Short Form Survey | SF-36 | 2 |
| Asthma Control Questionnaire | ACQ | 2 |
| Bath adolescent pain questionnaire | BAPQ | 2 |
| Caregiver Priorities and Child Health Index of Life with Disabilities | CP-QoL | 2 |
| Cerebral Palsy Quality of Life Questionnaire for Children | CPCHILD | 2 |
| Child Behaviour Checklist | CBCL | 2 |
| Child Health Questionnaire | CHQ | 2 |
| European Organization for the Research and Treatment of Cancer Quality of Life Questionnaire | EORTC-QLQ-30 | 2 |
| Functional Assessment of Chronic Illness Therapy - Fatigue | FACIT-F | 2 |
| Hip Outcome Score - Sports Specific Subscale | HHS | 2 |
| Hospital Anxiety and Depression Scale | GROC | 2 |
| KIDSCREEN-27 | K-SE | 2 |
| Participation and Environment Measure for Children and Youth | PEM-CY | 2 |
| Pediatric Motor Activity Log - How Often subscale | PMAL | 2 |
| Pediatric Quality of Life Inventory - Multidimensional Fatigue Scale | PedsQL-MFS | 2 |
| physical activity scale | PAS | 2 |
| Post-Concussion Symptom Scale | PCSS | 2 |
| Preferences for Activities of Children | PAC | 2 |
| Strengths and Difficulties Questionnaire | SDQ | 2 |
| Tampa Scale of Kinesiophobia | TSK | 2 |
| 12-Item Short Form Survey | SF-12 | 1 |
| activity diary | activity diary | 1 |
| Activity Questionnaire for Adults and Adolescents | AQuAA | 1 |
| ACTIVLIM | ACTIVLIM | 1 |
| aesthetic appearance visual analog scale | aesthetic appearance VAS | 1 |
| Anterior Cruciate Ligament Quality of Life instrument | ACl-QOL | 1 |
| Anterior Cruciate Ligament Return to Sport AfterInjury instrument | ACL-RSI | 1 |
| Asthma Control Test | ACT | 1 |
| Athlete Fear Avoidance Questionnaire | AFAQ | 1 |
| averange defecation frequency | DF | 1 |
| Barriers to Participation in Physical Activities Questionnaire | BPPA-Q | 1 |
| Beck Anxiety Inventory | BAI | 1 |
| Beck Depression Inventory, second edition | BDI-II | 1 |
| Behavior Assessment System for Children | BASC | 1 |
| Belief in Goal Self-competence Scale | BiGSS | 1 |
| Body Perception Questionnaire | BPQ | 1 |
| Camper Confidence Scale | CSS | 1 |
| Child and Adolescent Scale of Participation | CASP | 1 |
| Child and Family Follow-up Survey | CFFS | 1 |
| Child Daily Questionnaire | CDQ | 1 |
| Child Health Utility–Nine Domains utility score | CHU9D | 1 |
| Child Simulator Sickness Questionnaire | SSQ | 1 |
| Child’s Somatization Inventory-24 | CSI-24 | 1 |
| Children’s Sleep Habits Questionnaire | CSHQ | 1 |
| Cincinnati knee rating system | CKRS | 1 |
| Concussion Symptom Endorsement | PCSI | 1 |
| Congenital Heart Disease-TNO/AZL Adult Quality of Life | CONHD-TAAQOL | 1 |
| Cumberland Ankle Instability Tool | CAIT | 1 |
| Cycling Skills Checklist | CSC | 1 |
| Cystic Fibrosis Questionnaire | CFQ | 1 |
| Dance Functional Outcome Survey | DFOS | 1 |
| Dimensions of Mastery Motivation Questionnaire | DMQ | 1 |
| Disabilities of the Arm, Shoulder, and Hand | DASH | 1 |
| Duruoz Hand Index | DHI | 1 |
| Face Scale Pain | FOPQ-C | 1 |
| fear thermometer | FSS | 1 |
| Foot and Ankle Ability Measure | FAAM | 1 |
| Forgotten Knee Joint Score | FIM | 1 |
| fucntioning visual analogue scale | FT | 1 |
| Gillette Functional Assessment Questionnaire | FAQ | 1 |
| Global rating of change | GOAL | 1 |
| Goal Attainment Scaling | functioning VAS | 1 |
| Harris Hip Score | HAID | 1 |
| Hip Outcome Tool | HOS-SSS | 1 |
| How Am I doing questionnaire | HADS | 1 |
| Injury–Psychological Readiness to Return to Sport scale | IPAQ-SF | 1 |
| International Knee Documentation Committee | iHOT-33 | 1 |
| International Physical Activity Questionnaire short form | IKDC | 1 |
| joy visual analogue scale | joy VAS | 1 |
| KINDL questionnaire | KIDSCREEN-27 | 1 |
| Kujala score | Kujala | 1 |
| Life Habits Questionnaire | LIFE-H | 1 |
| linear analogue scale | LAS | 1 |
| Multidimensional Anxiety Scale for Children | MASC | 1 |
| Multidimensional Fatigue Scale | MFI | 1 |
| Multidimensional Pain Inventory | MPI | 1 |
| Neuroquality of Life-Stigma | Neuro-QoL Stigma | 1 |
| Nijmegen Questionnaire | NQ | 1 |
| Non-Arthritic Athletic Hip Score | NAAHS | 1 |
| Non-arthritic Hip Score | NAHS | 1 |
| Numeric Rating Scale for sleep | S-NRS | 1 |
| numeric rating scales assessing quality of life | NRS-QoL | 1 |
| OMNI Walk/Run rating of perceived exertion | OMNI-RPE | 1 |
| Oswestry Disability Index | ODI | 1 |
| Oxford Ankle and Foot Questionnaire | OAFQ | 1 |
| Pain Coping Questionnaire | PCQ | 1 |
| pain location map | pain LM | 1 |
| Pain Stages of Change Questionnaire | PSOCQ | 1 |
| Patient’s Global Impression of Change | PGIC | 1 |
| Patient-Reported Outcomes Measurement Information System - Anxiety | PROMIS-A | 1 |
| Patient-Reported Outcomes Measurement Information System - Cognitive Functioning | PROMIS-CF | 1 |
| Patient-Reported Outcomes Measurement Information System - Depressive Symptoms | PROMIS-DS | 1 |
| Patient-Reported Outcomes Measurement Information System - Fatigue | PROMIS-F | 1 |
| Patient-Reported Outcomes Measurement Information System - Mobility | PROMIS-M | 1 |
| Patient-Reported Outcomes Measurement Information System - Pain Behavior | PROMIS-PB | 1 |
| Patient-Reported Outcomes Measurement Information System - Pain Interference | PROMIS-PI | 1 |
| Patient-Reported Outcomes Measurement Information System - Peer Relations | PROMIS-PR | 1 |
| Patient-Reported Outcomes Measurement Information System - Upper Extremity | PROMIS-UE | 1 |
| Patient-Reported Outcomes Measurement Information System Profile 49 | PROMIS-49 | 1 |
| Pediatric Cardiac Quality of Life Inventory | PCQLI | 1 |
| Pediatric Migraine Disability Assessment tool | PedMIDAS | 1 |
| Pediatric Pain Screening Tool | PPST | 1 |
| Pediatric Quality of Life Inventory - Cancer Module | PedsQL-C | 1 |
| Pediatric Quality of Life Inventory - Cerebral Palsy Module | PedsQL-CP | 1 |
| Pediatric Quality of Life Inventory - Family Impact Module | PedsQL-FIM | 1 |
| Pediatric Quality of Life Inventory - Gastrointestinal symptoms scale | PedsQL-GSS | 1 |
| Pediatric Quality of Life Inventory - Health Satisfaction Module | PedsQL-HS | 1 |
| Personality Inventory for Children | PIC | 1 |
| Photograph Series of Daily Activities for youth | PHODA-youth | 1 |
| Physical Activity Questionnaire | PAQ | 1 |
| Pictorial Scale of Movement Skill Competence | PMSC | 1 |
| Piers’ Harris Children’s Self-Concept Scale | Piers-Harris | 1 |
| Post-Concussion Symptom Inventory | PCS-R | 1 |
| satisfaction with treatment visual analogue scale | satisfaction with treatment VAS | 1 |
| Scoliosis Research Society-22r | SRS-22r | 1 |
| self-perception visual analogue scale | self-perception VAS | 1 |
| Short Falls Efficacy Scale | sFES | 1 |
| Short Sensory Profile | sSP | 1 |
| Spinal Appearance Questionnaire | SAQ | 1 |
| Structural analysis of social behavior | SASSB | 1 |
| Symptom Severity Score | SSS | 1 |
| The Fear of Pain Questionnaire-Child report | FJS | 1 |
| TNO/AZL Child Quality of Life Questionnaire | TACQOL | 1 |
| Treatment Evaluation Inventory – Short Form | TEI-SF | 1 |
| Upper Extremity Functional Index | UEFI | 1 |
| verbal rating scale for pain | pain VRS | 1 |
| visual motivation tool with five faces | VMT | 1 |
| Wong-Baker Faces Pain Scale | WBFPS | 1 |
| World Health Organization Disability Assessment Schedule | WHODAS | 1 |
| World Health Organization Quality of Life Brief Version | WHOQOL-BREF | 1 |
| ZL/TNO Questionnaire Quality of Life | DUX-25 | 1 |
