# Supplementary material for: Patient reported outcomes in pediatric physical therapy: a scoping review and evidence map
Source: J Patient Rep Outcomes. 2025 Oct 24;9:125. doi: 10.1186/s41687-025-00947-5 (PMC12552199; doi:10.1186/s41687-025-00947-5)
Supplement: Supplementary file 5 — Supplementary Material 5 [file 41687_2025_947_MOESM5_ESM.docx]

**Additional file 5: PROMs used per PRO in the included studies**

Important note: In this study, psychometric properties of patient reported outcome measures (PROMs) were not evaluated. Therefore, this table should not be used as guidance to choose a PROM for use, either in daily practice or in research.

| **Patient Reported Outcome (PRO)** | **Number of PROMs used to measure this PRO** | **PROMs used to measure the PRO (see** **Additional file 3 for list of abbreviations)** |
| --- | --- | --- |
| activities and participation | 34 | ABILHAND-Kids; ACTIVLIM; BiGSS; BPPA-Q; CAPE; CASP; CFFS; CHAQ; CHEQ; COPM; CP-QoL; DCD-Q; DHI; FIM; LIFE-H; Neuro-QoL Stigma; NPRS; PAC; PEDI; PEDI-CAT; PedsQL; PedsQL-CP; PEM-CY; PIC; PMAL; PODCI; PROMIS-CF; PROMIS-F; PROMIS-M; PROMIS-PB; PROMIS-PR; PROMIS-UE; PSFS; WBFPS |
| anxiety | 32 | ACl-QOL; ACL-RSI; AFAQ; BAI; BAPQ; CDI; CHQ; COPM; FDI; FOPQ-C; FSS; FT; IKDC; KOOS; K-SE; MASC; NPRS; pain VAS; PCS; PCSI; PEDI; PedsQL; PHODA-youth; PROMIS-49; PROMIS-A; PROMIS-DS; PSOCQ; self-created; sFES; sSP; TEI-SF; TSK |
| body image | 12 | aesthetic appearance VAS; BPQ; DASH; EQ-5D; GOAL; IPAQ-SF; pain VAS; PODCI; SAQ; SASSB; satisfaction with treatment VAS; SRS-22r |
| community,social and civic life | 5 | BiGSS; BPPA-Q; COPM; CP-QoL; PEM-CY |
| confidence | 8 | BASC; COPM; CP-QoL; CSS; GOAL; KINDL; PedsQL; self-created |
| defecation functions | 5 | BSFS; DF; PedsQL; PedsQL-GSS; SSS |
| depression | 24 | BAI; BAPQ; BDI-II; CBCL; CDI; CES-DC; CHAQ; CSHQ; FDI; FOPQ-C; FSS; MASC; NPRS; pain VAS; PCQ; PCS; PCS-R; PedsQL; PPST; PROMIS-49; PROMIS-A; PROMIS-DS; SPP; TEI-SF |
| education | 11 | BAPQ; BSFS; CDI; CES-DC; CSHQ; FDI; KINDL; NPRS; PCS; PedsQL; PedsQL-GSS |
| emotional functions | 15 | ACl-QOL; ACL-RSI; BAPQ; BSFS; CP-QoL; CSI-24; KINDL; K-SE; NPRS; OMNI-RPE; PCS; PCSI; PedsQL; PedsQL-GSS; PODCI |
| family relationships | 13 | BAPQ; CAPE; CFFS; CP-QoL; FIM; KINDL; PCS; PedsQL; PedsQL-FIM; PedsQL-HS; PIC; PODCI; WHOQOL-BREF; |
| fatigue | 28 | ABILHAND-Kids; AQuAA; BAI; Borg; CFFS; CHAQ; CHQ; CSI-24; EORTC-QLQ-30; FACIT-F; FIM; FSS; FT; GOAL; joy VAS; KINDL; MFI; NPRS; OMNI-RPE; pain LM; pain VAS; PCSI; PEDI; PedsQL; PedsQL-MFS; PROMIS-49; self-created; VMT |
| functions of the joints and bones | 4 | CKRS; KOOS; Kujala; pain VAS |
| functions of the respiratory system | 3 | ACQ; ACT; NQ |
| functions related to the digestive system | 3 | BSFS; PedsQL; PedsQL-GSS |
| general health perceptions | 9 | CONHD-TAAQOL; CP-QoL; DFOS; LAS; PEDI; PedsQL; PedsQL-CP; SF-36; TACQOL |
| general physical endurance | 4 | CDI; CHAQ; PCQ; PPST |
| general tasks and demands | 60 | ABILHAND-Kids; ACTIVLIM; CAIT; CAPE; CDI; CHAQ; CHQ; CHU9D; COPM; CSC; CSHQ; DCD-Q; DFOS; DHI; EORTC-QLQ-30; EQ-5D; FAAM; FACIT-F; FAQ; FDI; FJS; FOPQ-C; GAS; GOAL; GROC; IPAQ-SF; KOOS; MASC; MPI; Neuro-QoL Stigma; NPRS; PAC; pain VAS; PAQ; PCQ; PCS; PEDI; PEDI-CAT; PedsQL; PGIC; PHODA-youth; PMAL; PMSC; PODCI; PPST; PROMIS-A; PROMIS-CF; PROMIS-DS; PROMIS-F; PROMIS-M; PROMIS-PB; PROMIS-PR; PROMIS-UE; PSFS; PSOCQ; SAQ; SPP; SRS-22r; TEI-SF; WBFPS |
| global psychosocial functions | 10 | BSFS; CBCL; CDI; COPM; DF; MPI; PedsQL; SDQ; SPP; SSS |
| goal attainment | 24 | ABILHAND-Kids; BiGSS; BPPA-Q; CAPE; CHAQ; CHQ; CHU9D; COPM; CP-QoL; CSC; CSS; functioning VAS; GAS; GOAL; PAC; pain VAS; PAQ; PEDI; PEDI-CAT; PEM-CY; PGIC; PMSC; self-created; SPP |
| hand and arm use | 10 | ABILHAND-Kids; CASP; CHEQ; COPM; GAS; LEFS; NPRS; PEDI; PMAL; UEFI |
| intellectual functions | 3 | COPM; PCSI; PEDI-CAT |
| interpersonal interactions and relationships | 18 | BAPQ; BSFS; CAPE; COPM; CP-QoL; KINDL; LEFS; pain VRS; PCS; PEDI; PEDI-CAT; PedsQL; PedsQL-GSS; PIC; PROMIS-49; self-created; sFES; sSP |
| managing one's own activity level | 9 | CKRS; COPM; EORTC-QLQ-30; EQ-5D; FACIT-F; KOOS; Kujala; pain VAS; self-created |
| mental functions | 2 | PedsQL; SDQ |
| mobility | 26 | ABILHAND-Kids; CES-DC; CHU9D; COPM; CP-QoL; CSC; DFOS; FAQ; FDI; GAS; GOAL; LEFS; NPRS; pain VRS; PAQ; PCS; PEDI; PEDI-CAT; PedsQL; PedsQL-CP; PMSC; PODCI; PROMIS-49; self-created; SPP; UEFI |
| motivation | 11 | Borg; EQ-5D; FDI; IPAQ-SF; NPRS; pain LM; pain VAS; PSOCQ; SAQ; SRS-22r; VMT |
| neuromusculoskeletal and movement-related functions | 51 | activity diary; BAPQ; BSFS; CAPE; CDI; CDQ; CES-DC; CHAQ; CHU9D; COPM; CSC; DCD-Q; DF; DMQ; EQ-5D; FDI; GAS; HAID; HHS; IKDC; IPAQ-SF; KINDL; KOOS; K-SE; LEFS; NAHS; PAC; pain VAS; pain VRS; PAQ; PCS; PCSI; PEDI; PEDI-CAT; PedsQL; PedsQL-FIM; PedsQL-GSS; PedsQL-HS; PedsQL-MFS; PMSC; PODCI; PROMIS-49; PSFS; SAQ; self-created; self-perception VAS; SPP; SRS-22r; SSS; TSK; WHOQOL-BREF; |
| orientation to self | 20 | activity diary; BPQ; BSFS; CBCL; CDI; CHU9D; CSC; DCD-Q; GAS; HAID; PAQ; PCQLI; PEDI; PEDI-CAT; PedsQL; Piers-Harris; PMSC; SASSB; self-perception VAS; SPP |
| overall quality of life | 78 | ACl-QOL; ACL-RSI; BASC; BiGSS; Borg; BPPA-Q; BSFS; CAPE; CBCL; CDI; CFFS; CFQ; CHAQ; CHQ; CHU9D; CKRS; CONHD-TAAQOL; COPM; CPCHILD; CP-QoL; CSC; DF; DUX-25; EORTC-QLQ-30; EQ-5D; FACIT-F; FIM; FJS; FPS; FT; GAS; GROC; iHOT-33; IKDC; IPAQ-SF; KIDSCREEN-27; KINDL; KOOS; K-SE; Kujala; LAS; MFI; NPRS; NRS-QoL; OAFQ; pain VAS; PAQ; PAS; PCQLI; PCS; PCSI; PEDI; PEDI-CAT; PedMIDAS; PedsQL; PedsQL-C; PedsQL-CP; PedsQL-FIM; PedsQL-HS; PEM-CY; PGIC; PIC; Piers-Harris; PMSC; PODCI; PROMIS-PI; SAQ; SDQ; self-created; SF-36; sFES; SPP; SRS-22r; sSP; SSS; TACQOL; TSK; WHOQOL-BREF; |
| pain | 77 | aesthetic appearance VAS; AFAQ; BAI; BAPQ; Borg; BSFS; CDI; CDQ; CES-DC; CHAQ; CHQ; CKRS; COPM; CP-QoL; CSHQ; CSI-24; DASH; DCD-Q; DFOS; DHI; EQ-5D; FDI; FJS; FOPQ-C; FPS; FSS; functioning VAS; GAS; GOAL; GROC; HADS; HHS; HOS-SSS; IKDC; KIDSCREEN-27; KOOS; K-SE; Kujala; LEFS; MASC; NAAHS; NAHS; NPRS; OAFQ; ODI; OMNI-RPE; pain LM; pain VAS; pain VRS; PAS; PCQ; PCS; PEDI; PedMIDAS; PedsQL; PedsQL-CP; PedsQL-MFS; PGIC; PHODA-youth; PODCI; PPST; PROMIS-49; PROMIS-A; PROMIS-DS; PROMIS-PI; PSFS; PSOCQ; satisfaction with treatment VAS; self-created; SF-12; S-NRS; SSQ; TEI-SF; TSK; UEFI; VMT; WBFPS |
| perceptual functions | 5 | BASC; COPM; PedsQL; sFES; sSP |
| recreation and leisure | 18 | activity diary; CAPE; COPM; CSS; DCD-Q; EORTC-QLQ-30; FACIT-F; FJS; HAID; IKDC; KOOS; K-SE; NPRS; PCS; PEDI; self-perception VAS; SPP; TSK |
| regulating behaviours within interactions | 4 | CBCL; CDI; PedsQL; SPP |
| self-care | 7 | ABILHAND-Kids; COPM; CSS; LEFS; pain VRS; PEDI; self-created |
| sensory functions | 5 | BDI-II; FPS; PCS-R; PCSS; SSQ |
| services, systems and policies | 2 | CP-QoL; GOAL |
| sleep functions | 6 | CDI; CSHQ; FDI; NPRS; PedsQL; S-NRS |
| sports | 15 | CAIT; EQ-5D; FAAM; FJS; GROC; IKDC; KOOS; K-SE; NPRS; pain VAS; PAS; PCS; PODCI; self-created; TSK |
| treatment expectation | 7 | CDI; FOPQ-C; MASC; PCS; PROMIS-A; PROMIS-DS; TEI-SF |
| undertaking mulitple tasks independently | 19 | ACl-QOL; ACL-RSI; BASC; BiGSS; BPPA-Q; CAPE; COPM; CP-QoL; GAS; GOAL; K-SE; LEFS; PAC; pain VRS; PEDI; PEDI-CAT; PedsQL; PEM-CY; self-created |
